# Genome-Wide Association Studies Identify the Loci for 5 Exterior Traits in a Large White × Minzhu Pig Population

**DOI:** 10.1371/journal.pone.0103766

**Published:** 2014-08-04

**Authors:** Ligang Wang, Longchao Zhang, Hua Yan, Xin Liu, Na Li, Jing Liang, Lei Pu, Yuebo Zhang, Huibi Shi, Kebin Zhao, Lixian Wang

**Affiliations:** 1 Key Laboratory of Farm Animal Genetic Resources and Germplasm Innovation of Ministry of Agriculture of China, Institute of Animal Science, Chinese Academy of Agricultural Sciences, Beijing, China; 2 Jilin Academy of Agricultural Sciences, Changchun, China; The University of Chicago, United States of America

## Abstract

As one of the main breeding selection criteria, external appearance has special economic importance in the hog industry. In this study, an Illumina Porcine SNP60 BeadChip was used to conduct a genome-wide association study (GWAS) in 605 pigs of the F_2_ generation derived from a Large White × Minzhu intercross. Traits under study were abdominal circumference (AC), body height (BH), body length (BL), cannon bone circumference (CBC), chest depth (CD), chest width (CW), rump circumference (RC), rump width (RW), scapula width (SW), and waist width (WW). A total of 138 SNPs (the most significant being MARC0033464) on chromosome 7 were found to be associated with BH, BL, CBC, and RC (*P*-value  = 4.15E-6). One SNP on chromosome 1 was found to be associated with CD at genome-wide significance levels. The percentage phenotypic variance of these significant SNPs ranged from 0.1–25.48%. Moreover, a conditional analysis revealed that the significant SNPs were derived from a single quantitative trait locus (QTL) and indicated additional chromosome-wide significant association for 25 SNPs on SSC4 (BL, CBC) and 9 SNPs on SSC7 (RC). Linkage analysis revealed two complete linkage disequilibrium haplotype blocks that contained seven and four SNPs, respectively. In block 1, the most significant SNP, MARC0033464, was present. Annotations from pig reference genome suggested six genes (*GRM4*, *HMGA1*, *NUDT3*, *RPS10*, *SPDEF* and *PACSIN1*) in block 1 (495 kb), and one gene (*SCUBE3*) in block 3 (124 kb). Functional analysis indicated that *HMGA1* and *SCUBE3* genes are the potential genes controlling BH, BL, and RC in pigs, with an application in breeding programs. We screened several candidate intervals and genes based on SNP location and gene function, and predicted their function using bioinformatics analyses.

## Introduction

External appearance is a major breeding selection criterion in the hog industry. Exterior traits such as body height (BH), body length (BL), cannon bone circumference (CBC), chest depth (CD), chest width (CW), and rump circumference (RC) are closely related to body growth, food intake capability, sow reproductive efficiency and longevity, and so on. Hence, these factors has attracted extensive attention in modern pig industry [1]. In human being, additive genetic effect explains 81% of the variation in height [2], and the heritability for BL,CD, and CW in swine are 0.16–0.32,0.34, and 0.25, respectively [1], [3]. Understanding the genetic mechanism of inter-individual variation in body measurements might provide new tools that can help manipulate animal growth and production [4].

In the past, advances in DNA-based marker technology made it possible to identify quantitative trait loci (QTLs) associated with complex economically important traits in pigs. To date, more than 762 QTLs for exterior traits have been identified via genome scans and included in the Pig QTLdb (http://cn.animalgenome.org/cgi-bin/QTLdb/SS/index) [5]. However, due to the large intervals of QTLs (covering more than 20 cM [6], even a whole chromosome [7]), few quantitative trait nucleotides (QTN) have been identified in pigs by fine-scale mapping of QTLs.

Since its emergence, high-density single nucleotide polymorphism (SNP) array has been widely used and proved to be a powerful tool in identification of causal mutations associated with complex traits such as reproduction, disease susceptibility, and meat quality in livestock [8], [9], [10], [11], [12], [13], [14]. Although numbers of genome-wide association studies (GWAS) have been carried out on pigs, only a few GWAS focused on identifying genes associated with exterior traits [1]. Moreover, among the exterior traits studied, GWAS data on important body measurement traits such as body height, cannon bone circumference, and rump circumference are still lacking.

In order to identify novel genes and novel quantitative trait nucleotides (QTN) related to exterior traits across the *Sus scrofa* genome, the present GWAS was performed to detect potential genetic variants associated with 10 exterior traits in a Large White × Minzhu pig resource population.

## Results

### Phenotype statistics

Summary statistics results of the phenotypes of the F_2_ individuals are presented in Table 1. The average abdominal circumference (AC), body height (BH), body length (BL), cannon bone circumference (CBC), chest depth (CD), chest width (CW), rump circumference (RC), rump width (RW), scapula width (SW), and waist width (WW) were 120.66 cm, 69.47 cm, 113.34 cm, 17.46 cm, 39.47 cm, 29.26 cm, 85.95 cm, 30.05 cm, 31.43 cm, and 26.95 cm, respectively. The phenotype and genetic correlations between each trait are shown in Table S1 in File S1. In the phenotype correlations analysis, CBC was highly correlated with BH and BL (correlation coefficients are 0.57 and 0.57, respectively), and was medium correlated with RC (correlation coefficients is 0.42). In the genetic correlations analysis, CBC was highly correlated with BH, BL, and RC (correlation coefficients are 0.55, 0.77 and 0.65, respectively).

**Table 1:** The description of the 10 analyzed traits.

Variable	N	Mean	Std Dev	Minimum	Maximum	Coeff of variation
**AC**	611	120.66072	7.939488	85	146	6.5800105
**BH**	612	69.474837	5.159726	35.6	81.5	7.4267548
**BL**	612	113.34199	9.558151	1.9	141	8.4330182
**CBC**	607	17.455848	1.64893	13.3	22.5	9.446292
**CD**	611	39.465466	2.63838	27.1	46.2	6.6852889
**CW**	612	29.262811	2.771028	1.55	36.8	9.4694526
**RC**	610	85.954262	7.908138	21	102	9.2004022
**RW**	549	30.049363	2.125064	22.6	35.9	7.0719115
**SW**	612	31.432516	2.634731	23.6	43.1	8.3821815
**WW**	612	26.95317	2.791757	18.3	37.9	10.357806

AC, abdominal circumference; BH, body height; BL body length; CBC, cannon bone circumference; CD, chest depth; CW, chest width; RC, rump circumference; RW, rump width; SW, scapula width; WW, waist width.

### GWAS and bioinformatics analysis

**Figure 1:**
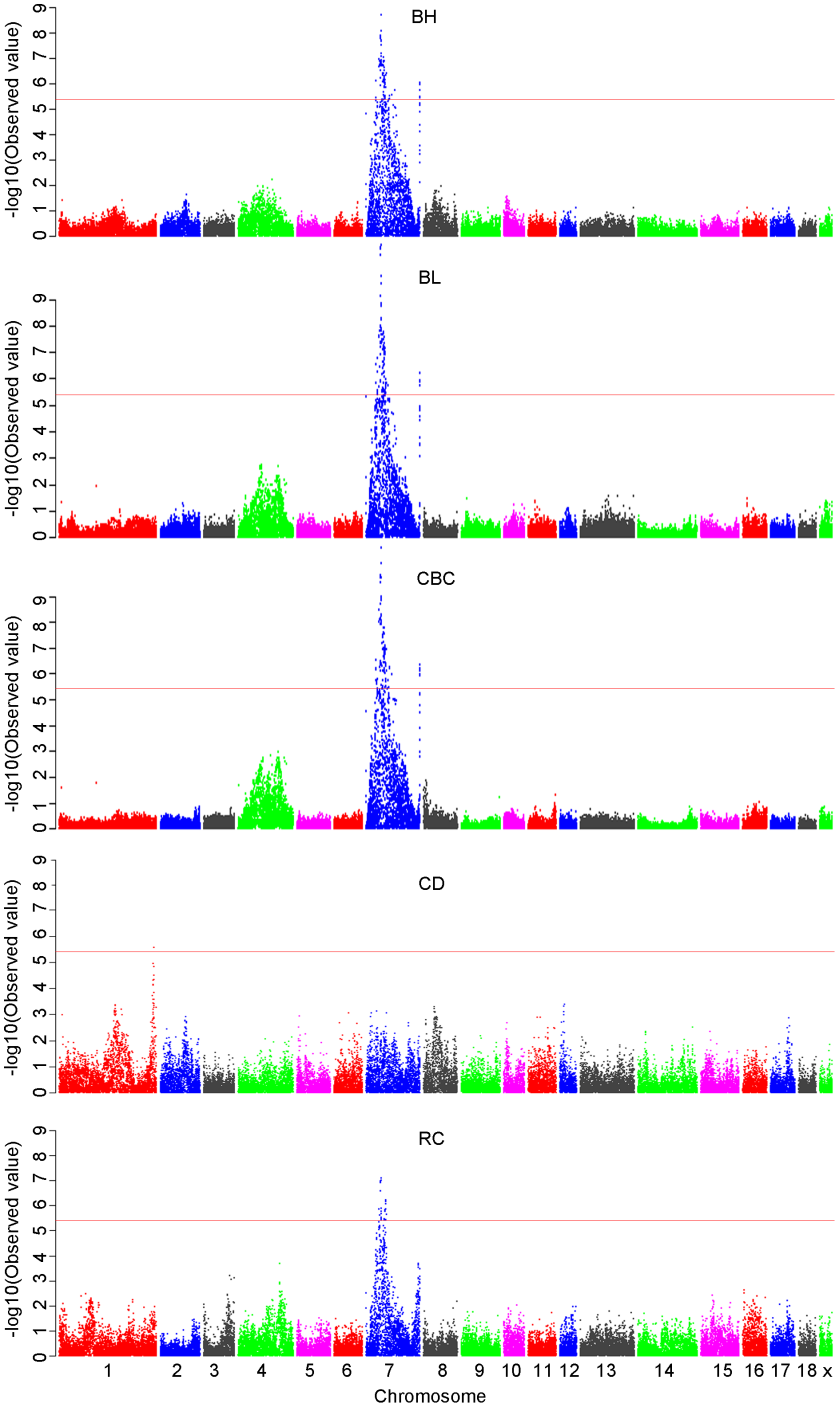
Manhattan plots of genome-wide association study with BH, BL, CBC, and RC. Chromosomes 1–18 and X are shown separated by color. The red horizontal lines indicate the genome-wide significance levels (-log_10_ (6.08E-05)).

**Figure 2:**
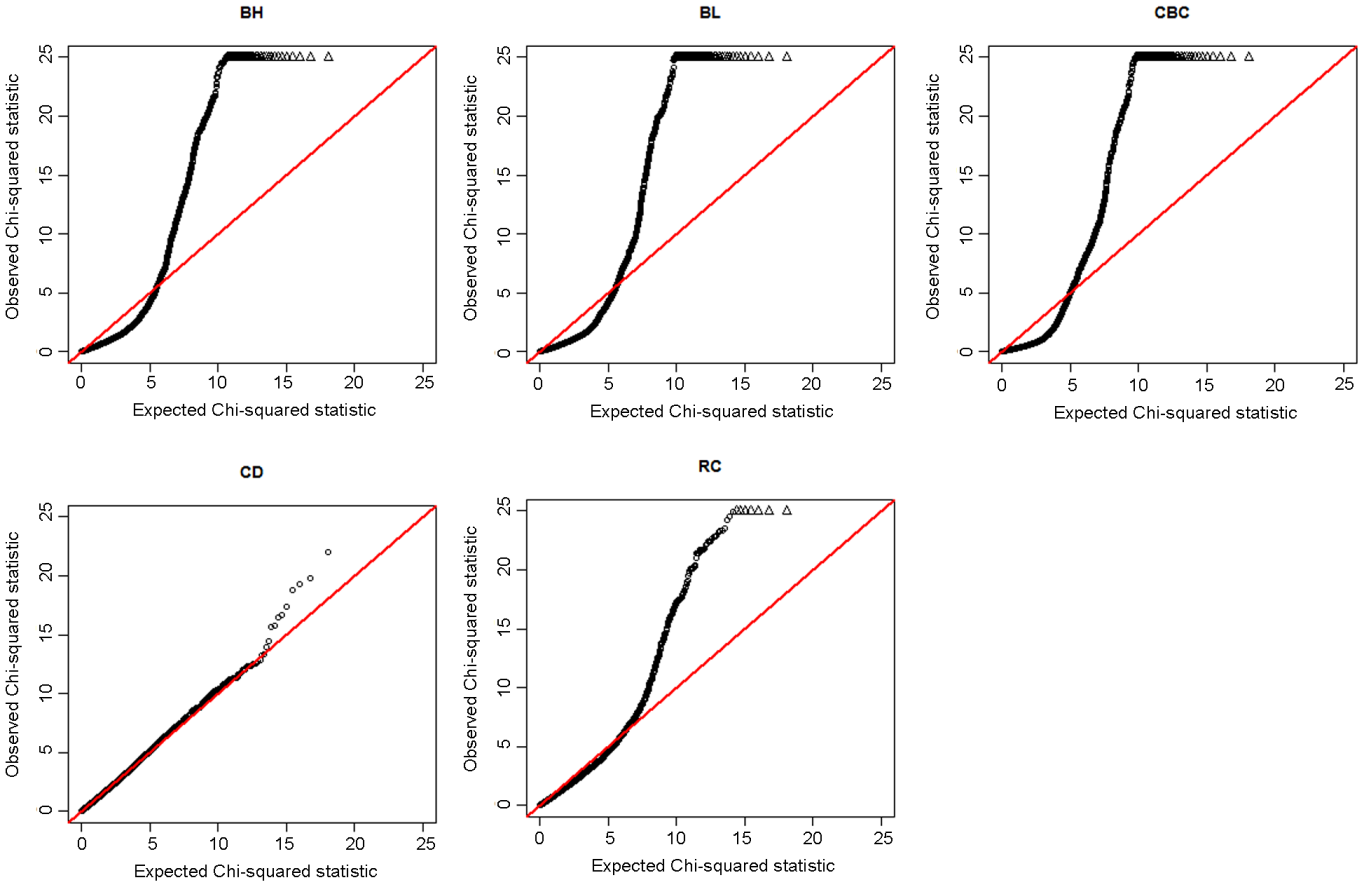
The Q-Q plots for body height (BH), body length (BL), cannon bone circumference (CBC), chest depth (CD) and rump circumference (RC). For each trait, Q-Q plot of the results derived without adjustment for the inflation factor (λ) are shown in black.

After the quality control procedure, 48,238 SNPs and 594 F_2_ individuals were used for the genome-wide association studies. The number of effective SNPs is 12309 and the significant threshold was 4.15E-06 (0.05/12039). None of the SNPs for AC, CW, RW, SW and WW reached a genome-wide significance level. One hundred and thirty-eight significant SNPs of BH, BL, CBC, and RC occurred in an enlarged region on SSC7 (Table S2 in File S1) while only one significant SNP of CD occurred on SSC1 (Table S3 in File S1). The Manhattan and quantile-quantile (Q-Q) plots for these five significantly associated traits are shown in Figure 1 and 2, The lambda value for BH, BL, CBC, RC and CD, were 1.18, 1.18, 1.18, 0.98, and 1.00, respectively. Other Manhattan plots and Q-Q plots are displayed in Figure S1 and S2 in File S2, respectively and the lambda value for other traits were closed to 1.The Q-Q plots and the lambda value indicated a certain degree of deviation between the predicted and actual data of BH, BL, and CBC.

Of the 138 SNPs on SSC7, 90, 115, 116, and 32 SNPs were significantly associated with BH, BL, CBC, and RC, respectively (Table S3 in File S1). The most significant SNP for each trait was MARC0033464, which defined 18.94%, 25.48%, 2.63% and 5.07% of the phenotypic variance for BH, BL, CBC, and RC, respectively (Table S4, S5, S6, S7 in File S1). All significant SNPs were BLASTed using Ensembl (http://asia.ensembl.org/index.html), and 25 SNPs were located within 24 known genes (Table 2). Using the online gene functional classification and annotation tools in the Database for Annotation, Visualization and Integrated Discovery (DAVID, http://david.abcc.ncifcrf.gov) [15], four statistically significant GO (Gene Ontology) [16] terms (P<0.05, Table S8 in File S1) were identified; however, none of the identified Kyoto Encyclopedia of Genes and Genomes (KEGG) pathways were significant [17]. The detected genes were *solute carrier family 26* (*SLC26A8*), *solute carrier family 22 member 7* (*SLC22A7*), primase, DNA, polypeptide 2 (*PRIM2*), and *molybdenum cofactor synthesis 1* (*MOCS1*) and the associated GO terms included organic anion transport, 4 iron, 4 sulfur cluster binding, iron-sulfur cluster binding, and metal cluster binding. Only one genome-wide significant SNP, H3GA0005226, in c-abl oncogene 1, non-receptor tyrosine kinase (*ABL1*) gene was located on SSC1.

**Table 2:** The description of the SNPs within genes.

SNP	Gene	Chr	Position^1^	Gene description
**DRGA0007323**	*LRRC16A*	7	21468020-21653398	leucine rich repeat containing 16A
**ALGA0039477**	*PGBD1*	7	24079744-24099490	Piggy Bac transposable element derived 1
**ALGA0039611**	*SLA-11*	7	26674362-26716913	Uncharacterized protein
**UMB10000108**	*CYP21*	7	27722776-27725977	sus scrofa cytochrome P450, family 21
**H3GA0020450**	*NEU1*	7	27853782-27858037	sialidase 1
**ASGA0032302**	*PRIM2*	7	32750198-33022968	primase, DNA, polypeptide 2
**ASGA0032322**	*BEND6*	7	33231047-33273159	BEN domain containing 6
**ALGA0040120**	*DST*	7	33504832-33751812	dystonin
**ALGA0040148**	*COL21A1*	7	33918801-34009970	collagen, type XXI, alpha 1
**ASGA0032562**	*UHRF1BP1*	7	35474279-35538654	Uncharacterized protein
**ALGA0040331**	*SLC26A8*	7	36660474-36714021	solute carrier family 26
**MARC0039406**	*C6orf89*	7	37451398-37483062	chromosome 6 open reading frame 89
**MARC0096194**	*PI16*	7	37504337-37514234	peptidase inhibitor 16
**DIAS0004695**	*MTCH1*	7	37520856-37539035	mitochondrial carrier 1
**DIAS0000130**	*BTBD9*	7	38947185-39089845	BTB (POZ) domain containing 9
**DIAS0000369**	*DNAH8*	7	39286129-39569290	dynein, axonemal, heavy chain 8
**MARC0001110**	*DNAH8*	7	39286129-39569290	dynein, axonemal, heavy chain 8
**ALGA0040640**	*DAAM2*	7	40365126-40413588	dishevelled associated activator of morphogenesis 2
**M1GA0010112**	*MOCS1*	7	40416828-40451199	molybdenum cofactor synthesis 1
**ALGA0040805**	*FOXP4*	7	42055648-42088654	forkhead box P4
**INRA0025193**	*CUL9*	7	43726808-43763816	cullin 9
**DBNP0001311**	*SLC22A7*	7	43828818-43835223	Sus scrofa solute carrier family 22, member 7
**H3GA0021272**	*TMEM151B*	7	45137721-45145865	transmembrane protein 151B
**DBMA0000241**	*AARS2*	7	45170562-45183626	alanyl-tRNA synthetase 2, mitochondrial
**MARC0028399**	*FSD2*	7	57653600-57679877	fibronectin type III and SPRY domain containing 2

1 Derived from *Sus scrofa* Build 10.2.

Chr: Chromosome

There are also peeks on other chromosomes, e.g. peeks on chromosome 4 for BH, BL and CBC (Figure 1). To avoid the potential missing of important SNPs, and meanwhile to investigate the LD SNPs of the most significant SNPs, most significant SNPs (MARC0033464 of BH, BL, CBC and RC, while H3GA0005226 of CD) were used for the conditional GWAS analysis. The Manhattan plots obtained from the conditional analysis are shown in Figure S3 in File S2. No other significant association of SNPs was detected after the conditional analysis. However, 25 SNPs on SSC4 (*P*-value <6.08E-05) and 9 SNPs on SSC7 (*P*-value <6.16E-05) showed chromosome-wide association with BL, CBC, and RC (Table S9 in File S1).

**Figure 3:**
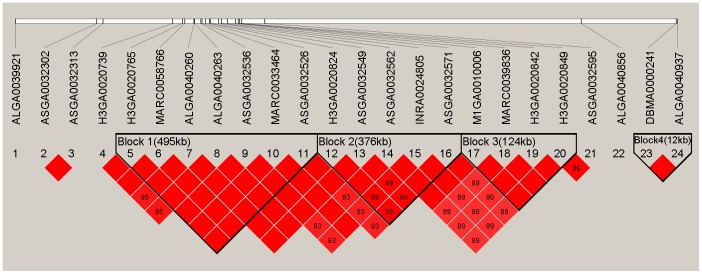
Haplotype block at linkage disequilibrium (LD) on a 4.76 Mb region on SSC7. Solid lines mark the three blocks identified. The block1 of 495(LD, r^2^ = 1).

Linkage analysis of a 4.76 Mb (from ALGA0039921 to H3GA0020849) region on SSC7 of significance (Table 3) that contained the 20 most significantly overlapping SNPs (Table 3) of BH, BL, CBC and RC revealed regions of complete LD (r^2^ = 1) haplotype block (block 1 and 3) (Figure 3). Moreover, block 1 with 495 kb (from H3GA0020765 to ASGA0032526) contained the most significant SNP MARC0033464. Haplotype frequencies were calculated and an association analysis was performed for block 1 and 3 with BH, BL, CBC, and RC (Table 4). The AAAGCAG (25.65%, with positive effect) and CGGAAGA (52.81%, with negative effect) haplotypes of block 1 were significantly associated with BH, BL, and RC (*P*<1E-5). The 495 kb haplotype included 6 annotated genes, which were *GRM4* (glutamate receptor, metabotropic 4), *HMGA1* (high mobility group AT-hook 1), *NUDT3* (nudix-type motif 3), *RPS10* (ribosomal protein S10), *SPDEF* (SAM pointed domain containing ETS transcription factor), and *PACSIN1* (protein kinase C and casein kinase substrate in neurons 1). The GGAA (33.14%, with positive effect) and AAGG (50.98%, with negative effect) haplotypes of block 3 were also significantly associated with BH, BL, and RC (*P*<1E-5). This 124 kb haplotype included 1 annotated gene named signal peptide CUB EGF-like 3 (*SCUBE3*).

**Table 3:** The detailed information about candidate regions and the most significant SNPs associated with BL, BH, CBC and RC.

name	Chr	Pos^1^	BL	BH	CBC	RC
**ALGA0039921**	7	31237418	2.46E-08	2.16E-07	9.74E-09	1.34E-06
**ASGA0032302**	7	32957768	1.59E-08	1.35E-07	8.02E-09	2.4E-06
**ASGA0032313**	7	33086096	1.59E-08	1.77E-07	7.78E-09	2.5E-06
**H3GA0020739**	7	34556148	2.2E-11	1.43E-08	2.84E-10	2.67E-07
**H3GA0020765**	7	34755602	1.12E-11	1.3E-08	1.57E-10	1.03E-07
**MARC0058766**	7	34803564	1.3E-11	1.74E-08	1.84E-10	1.25E-07
**ALGA0040260**	7	35002839	1.45E-09	8.25E-08	8.64E-09	3.23E-06
**ALGA0040263**	7	35017672	1.45E-09	8.25E-08	8.64E-09	3.23E-06
**ASGA0032536**	7	35150544	1.45E-09	8.25E-08	8.64E-09	3.23E-06
**MARC0033464**	7	35177641	8.94E-12	2.01E-09	1.31E-11	7.97E-08
**ASGA0032526**	7	35251345	1.45E-09	8.25E-08	8.64E-09	3.23E-06
**H3GA0020824**	7	35332373	2.06E-08	1.17E-07	5.69E-09	1.74E-06
**ASGA0032549**	7	35356274	2.06E-08	1.17E-07	5.69E-09	1.74E-06
**ASGA0032562**	7	35530333	6.12E-09	2.08E-08	1.28E-09	3.77E-06
**INRA0024805**	7	35579961	5.59E-09	2.2E-08	1.44E-09	3.15E-06
**ASGA0032571**	7	35709335	6.12E-09	2.14E-08	1.38E-09	2.96E-06
**M1GA0010006**	7	35880196	6.24E-09	6.71E-08	5.28E-09	2.13E-06
**MARC0039836**	7	35935629	1.33E-10	2E-08	1.90E-10	9.84E-08
**H3GA0020842**	7	35959385	1.83E-09	3.18E-08	1.07E-09	1.25E-06
**H3GA0020849**	7	36004578	1.35E-10	2.21E-08	1.99E-10	9.92E-08

1 Derived from *Sus scrofa* Build 10.2.

BH, body height; BL body length; CBC, cannon bone circumference; CD, chest depth; RC, rump circumference.

**Table 4:** Haplotype association analysis of Block 1 and Block 3 with BH, BL, CBC and RC.

Traits	Hap	Global p-val^1^	Type	SNP	Hap-Freq^2^	Hap-Score^3^	p-val^4^
**BH**	Block1	<1E-5	Haplo1	CGGAAGA	0.52805	−8.36169	<1E-5
			Haplo2	AAAGCAG	0.29651	10.40761	<1E-5
	Block3	<1E-5	Haplo1	AAGG	0.50977	−8.40519	<1E-5
			Haplo2	GGAA	0.3314	9.77698	<1E-5
**BL**	Block1	<1E-5	Haplo1	CGGAAGA	0.52805	−9.1758	<1E-5
			Haplo2	AAAGCAG	0.29651	12.02618	<1E-5
	Block3	<1E-5	Haplo1	AAGG	0.50977	−9.6648	<1E-5
			Haplo2	GGAA	0.3314	11.84945	<1E-5
**CBC**	Block1	0.01378	Haplo1	CGGAAGA	0.52805	−2.82456	0.00473
			Haplo2	AAAGCAG	0.29651	3.13383	0.00173
	Block3	0.00351	Haplo1	AAGG	0.50977	−2.9837	0.00285
			Haplo2	GGAA	0.3314	3.23023	0.00124
**RC**	Block1	1E-05	Haplo1	CGGAAGA	0.52805	−3.99267	7E-5
			Haplo2	AAAGCAG	0.29651	5.15674	<1E-5
	Block3	<1E-5	Haplo1	AAGG	0.50977	−4.33596	1E-5
			Haplo2	GGAA	0.3314	5.19853	<1E-5

1 The overall association between haplotypes and the response.

2 Estimated frequency of each haplotype in the population.

3 The score for the haplotype, which is the statistical measurement of association of each specific haplotype with the trait.

4 The asymptotic chi-square *P*-value was calculated from the square of the score statistic.

## Discussion

A GWAS using the PorcineSNP60 BeadChip was performed by Mix Model and Regression-Genomic Control (GRAMMAR-GC) methods. This pedigree-based method could exploit inter-family variation in addition to intra-family variation, and could rapidly analyze hundreds of thousands of markers [18], [19]. Although the genomic control (GC) procedure was used to correct for stratification, we also could see a little population stratification of some of the traits. We think the population stratification is odd and maybe caused by cryptic relatedness.

Heavy linkage disequilibrium in intercross population is one of the well-known limitations in genetic studies, which makes it difficult to identify the causative gene and mutation. In order to decrease non-positive rates, Bonferroni corrections have been used for all multiple tests. Since the Bonferroni correction is a conservative method to determine significance thresholds, we calculated the number of effective SNPs, and used the number of effective SNPs for the correction to ensure that no QTL was missed. Anyhow, there are no SNP significant associated with AC, CW, RW, SW and WW in present study. This may suggest that single locus plays a minor role in AC, CW, RW, SW and WW variation or the variation of AC, CW, RW, SW and WW were caused by copy number variation and so on.

In our research, the genome-wide significant association of SNPs on SSC7 disappeared after conditional analysis (Figure S1 in File S2 and Table S9 in File S1). This may suggest that these significant SNPs are derived from a single QTL.

It is known that one QTL could influence a multifactorial trait and most biological traits also have a complex inheritance influenced by numerous genes [20]. In the phenotype and genetic correlation analysis in the present study, the BH, BL, CBC and RC showed high phenotype or genetic correlations with each other suggested significant association between the major genes responsible for these traits. This GWAS also showed that all significant SNPs associated with more than one exterior trait localized in the same region, suggesting a pleiotropic effect of QTL on SSC7. Therefore, we chose the 20 most significant SNPs for further analysis. These SNPs, which were associated with every trait, are located in the proximal region from 31.24 Mb to 36.00 Mb on SSC7. SSC7 has been previously reported to be rich in QTLs influencing exterior traits [21], [22]. The region from 17.05 Mb to 45.42 Mb on SSC7 has been shown to have pleiotropic and significant QTL effects on CBC and Body length, measured in a Duroc purebred population and a White Duroc × Erhualian F2 intercross population [21], [22], [23], [24], [25].

In previous reports, QTLs detected in F2 populations usually defined a high percentage of phenotypic variance [16], [19]. Similar to these reports, the present study indicated that 18.94% and 25.48% phenotypic variance of BH and BL, respectively, were explained by QTLs, which suggests that QTLs have a major effect on BH and BL in this 4.76 Mb region.

Linkage analysis revealed two haplotype blocks in the significant region on SSC7 at complete linkage disequilibrium, which contained eight annotated genes of the pig reference genome. Of the 8 genes, *HMGA1* is of particular interest because it is ubiquitous in all cells of higher eukaryotes and the *HMGA1* protein plays an important role in cell growth and differentiation [26], [27]. By influencing the expression of two *IGFBP* (insulin-like growth factor binding protein) protein species, *HMGA1* serves as a *IGF1* (modulator of insulin-like growth factor 1) activity [28]. Comparing to its abundance during embryonic development, the *HMGA1* protein is nearly disappeared in fully differentiated adult tissues [29]. A single SNP haplotype of *IGF1* is usually present in small dog breeds, but nearly vanished in giant breeds, indicating that the variant plays important role in body size [30]. Moreover, in human studies, some GWAS have reported that *HMGA1* may be an ideal candidate as an anthropometric trait [31], [32], [33]. For instance, *HMGA1* might be a prime candidate for BH and BL in pigs.

*SCUBE3* (signal peptide CUB EGF-like 3) is also of particular interest. *SCUBE* proteins usually have three domains including multiple epidermal growth factor (EGF) domains, bone morphogenetic protein 1 (BMP1) C-terminal complement subcomponent (CUB) domain, and a huge spacer domain with several N-linked glycosylation sites [34]. In humans, *SCUBE3* functions as an endogenous transforming growth factor (*TGFβ*) receptor ligand, increasing Smad2/3 phosphorylation, and thus up-regulates target genes such as hedgehog (*Hh*), *TGFβ*, and bone morphogenetic protein 2 (*BMP2*) [35]. In human primary osteoblasts and long bones such as humerus and femur, the expression level of *SCUBE3* is usually very high [36], indicating that this gene is a major contributor to bone cell development. Thus, *SCUBE3* might also be an influencing factor in determining the size of bones and is a prime candidate for BH, BL, and RC in pigs.

In summary, this study identified a total of 138 significant SNPs on SSC7 associated with one or more exterior traits and one SNP on SSC1 associated with CD. Conditional analysis indicated that significant SNPs originated from a single QTL. Furthermore, linkage analysis identified two complete linkage disequilibrium regions containing the *HMGA1* and *SCUBE3* gene. Bioinformatics analysis indicated that *HMGA1* and *SCUBE3* genes might be prime candidates for some exterior traits in pigs.

## Materials and Methods

### Ethics statement

All animals used in the study were housed and handled following the guidelines of experimental animals established by the Council of China. Animal experiments were approved by the Science Research Department of the Institute of Animal Science, Chinese Academy of Agricultural Sciences (CAAS) (Beijing, China).

### Animals and phenotypic data

A 3-generation resource population constructed by intercrossing Large White boars and Minzhu sows between 2007 and 2011 was used in this study. In F1 generation, four Large White boars were mated with 16 Minzhu sows. Nine boars and 46 sows, all from the F1 population, were intercrossed to produce an F2 population consisted of 618 animals. All the animals were reared under the same nutritional and environmental conditions, and were housed at the experiment base of Institute of Animal Science, CAAS. All ten traits were measured by following standard procedures. Phenotypic and genetic correlation**s** among the traits were calculated to investigate whether they reflect the correlation among GWAS results. Pearson and genetic correlations among the traits were performed using SAS (version 9.1) and DMU (version 4.7), respectively.

### Genotyping and data quality control

A very small piece of ear tissue (about 50 mg) was collected when the ear number were coded using the ear tag pliers, and the l*ongissimus dorsi* were collected when the pigs were dead, all the procedure need no anaesthesia or analgesia disposition. Genomic DNA was extracted from the ear or *longissimus dorsi* using the phenol-chloroform method. Quantification and qualification of DNA were performed using a NanoDrop 2000 (Thermo Fisher Scientific Inc., Wilmington, DE, USA) with the standard: DNA concentration >50 ng/ul; 260/280 ratio between 1.8 and 2.1; 260/230 ratio >1.8. Genotyping was performed using the Illumina Porcine SNP60 BeadChip containing 62,163 SNPs across the whole genome. All F2 animals were genotyped using the BEADSTUDIO software (version 2.0, Illumina). Before quality control, the maximum likelihood method was applied using the Cervus program [37] to check pedigree mismatching using SNP information. F2 animals were quality controlled by the GenABEL R package. In the quality control procedure, call rate (CR) more than 90%, genotype minor allele frequency (MAF) more than 3% and Hardy-Weinberg equilibrium (HWE) values (*P*<10-6) were applied.

### Statistical analysis

The association analysis was conducted using the Mix Model and Regression - Genomic Control (GRAMMAR-GC) method [18], [19].

In the first step, the residuals were analyzed using DMU software.

In this step, data were analyzed using the mixed model:
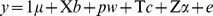
where *y* is the vector of phenotypes of all F2 individuals, *b* is the vector of fixed effects (consisting of the sex, parity and herd-year-season effect), *w* is the vector of body weights of the individuals (considered as a covariate), *c* is the vector of random effect (litter, c∼N(0, σ_c_^2^), *α* is the vector of random additive genetic effects with *α*∼N(0, Aσ_α_^2^) (where A is the relationship matrix calculated from the corrected pedigree and σ_α_^2^ is the additive genetic variance), X, T and Z are incidence matrices relating records in *y* to fixed and random effects, *p* is the regression coefficient of body weight and *e* is the vector of residual errors with *e*∼N(0, Iσ*_e_* ^2^),where *I* is the identity matrix and σ*_e_* ^2^ is the residual variance.

The vector of residuals y^*^ is estimated as
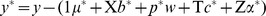
where b^*^, p^*^, c^*^ and α^*^are estimates and predictors for *b*, *p*, *c* and *α*, respectively.

Second, the residuals were used as the dependent traits for single locus regression analysis, and the unadjusted test statistic factor of the i^th^ SNP T_i_^2^ was calculated in the genomic control (GC) procedure in the R statistical environment using the GenABEL package [18], [19].

The Bonferroni method was employed for the genome-wide significance threshold, in which the conventional *P*-value was divided by the number of effective SNPs. The number of genome-wide effective SNPs, which was 12,039 (Table S2, File S1), was estimated using a simpleM method [38] following 3 steps: 1) Using the *cor* () function in R to derive the CLD correlation matrix from the SNP data set; 2) Using the R function *eigen* () to calculate the eigenvalues and 3) Inferring effective number of independent tests (Meff G) through PCA.

The significant threshold was 4.15E-06 (0.05/12039). The Manhattan plot was constructed using the GAP package and Q-Q plot was constructed within the R statistical environment. Proportion of the explained variation of each SNP is calculated using Tassel software [39].

### Conditional GWAS

In the conditional GWAS, the most significant SNP for each trait was used as a additional fixed effect, and all the analysis were performed by following the state GWAS procedure. The most significant SNP, MARC0033464, was used for the conditional analysis of BH, BL, CBC, and RC, while the most significant SNP H3GA0005226 was used for conditional analysis of CD.

### Bioinformatics analysis

All significant SNPs were BLASTed in the websites of Ensembl and NCBI (http://www.ncbi.nlm.nih.gov/). Genes within the detected regions were retrieved from the Ensembl Genes 64 Database using the BioMart software (http://www.biomart.org). Gene ontology and KEGG pathway analyses were performed to extract the functional annotation clustering and the pathways involved using DAVID (http://david.abcc.ncifcrf.gov) with the threshold of P<0.05.

### Haplotype analysis

Haplotype block detection was performed in the region which contained the 24 overlapped SNPs that were most significantly associated with the selected traits. The HAPLOVIEW V3.31 program [40] was used to detect and visualize the haplotype blocks with default parameters. The genotypes of significant SNP loci for 594 F2 individuals and their parents were used to detect the haplotype blocks.

Association analysis of the detected haplotype blocks and traits of 594 F2 individuals were performed using the Haplo.Stats R package [41]. The global score statistic index, positive/negative score for a particular haplotype (Hap-Score) *P*-value for the significance of each hap-score (p-val) and the frequency of each haplotype (Hap-Freq) were calculated to test overall associations among haplotype blocks and traits.

## Supporting Information

File S1 **Table S1. The correlations between each trait. Table S2. Distribution of SNPs and effective SNPs after quality control. Table S3. Genome – wide significant SNPs of BH, BL, CBC, and RC. Table S4. Percentage of phenotypic variance explanation of BH. Table S5. Percentage of phenotypic variance explanation of BL. Table S6. Percentage of phenotypic variance explanation of CBC. Table S7. Percentage of phenotypic variance explanation of RC. Table S8. Gene Ontology of 24 genes. Table S9. Chromosome-wide significant SNPs of BH, BL, CBC, and RC after conditional GWAS.**
(DOCX)

File S2 **Figure S1. The Manhattan plots for AC, CW, RW, SW and WW. Figure S2. The Q-Q plots for AC, CW, RW, SW and WW. Figure S3. The Manhattan plots for conditional GWAS for BH, BL, CBC, CD and RC.**
(DOCX)
