# Supplementary material for: Genome-Wide Association Studies Identify the Loci for 5 Exterior Traits in a Large White × Minzhu Pig Population
Source: PLoS One. 2014 Aug 4;9(8):e103766. doi: 10.1371/journal.pone.0103766 (PMC4121205; doi:10.1371/journal.pone.0103766)
Supplement: File S1 — Table S1. The correlations between each trait. Table S2. Distribution of SNPs and effective SNPs after quality control. Table S3. Genome – wide significant SNPs of BH, BL, CBC, and RC. Table S4. Percentage of phenotypic variance explanation of BH. Table S5. Percentage of phenotypic variance explanation of BL. Table S6. Percentage of phenotypic variance explanation of CBC. Table S7. Percentage of phenotypic variance explanation of RC. Table S8. Gene Ontology of 24 genes. Table S9. Chromosome-wide significant SNPs of BH, BL, CBC, and RC after conditional GWAS. (DOCX) [file pone.0103766.s001.docx]

**Table S1. The correlations between each trait**

|  | AC | BH | BL | CBC | CD | CW | RC | RW | SW | WW |
| --- | --- | --- | --- | --- | --- | --- | --- | --- | --- | --- |
| AC | 1.0000 | 0.3675 | 0.3036 | 0.2667 | 0.7410 | 0.6991 | 0.3379 | 0.6044 | 0.6515 | 0.5286 |
| BH | 0.8681 | 1.0000 | 0.4721 | 0.5736 | 0.4781 | 0.1659 | 0.3338 | 0.2412 | 0.2347 | 0.0994 |
| BL | 0.4546 | 0.7448 | 1.0000 | 0.5656 | 0.3793 | 0.2062 | 0.3393 | 0.2004 | 0.1751 | 0.0518 |
| CBC | 0.4145 | 0.5542 | 0.7712 | 1.0000 | 0.3453 | 0.1160 | 0.4199 | 0.2017 | 0.1943 | -0.0660 |
| CD | 0.866 | 0.9184 | 0.7269 | 0.7397 | 1.0000 | 0.5114 | 0.3893 | 0.4681 | 0.5806 | 0.4203 |
| CW | 0.4005 | -0.1041 | -0.4513 | -0.2351 | 0.0173 | 1.0000 | 0.2820 | 0.6970 | 0.6836 | 0.3643 |
| RC | 0.4743 | 0.2787 | 0.4291 | 0.6514 | 0.4471 | 0.433 | 1.0000 | 0.3371 | 0.3584 | 0.1829 |
| RW | 0.3534 | -0.0619 | -0.246 | -0.0959 | -0.0418 | 0.8448 | 0.6573 | 1.0000 | 0.7019 | 0.6760 |
| SW | 0.6557 | 0.2807 | 0.0412 | 0.416 | 0.479 | 0.7689 | 0.8105 | 0.7622 | 1.0000 | 0.5910 |
| WW | 0.4677 | 0.1621 | 0.1187 | 0.2617 | 0.2038 | 0.6563 | 0.8748 | 0.9218 | 0.8128 | 1.0000 |

AC, abdominal circumference; BH, body height; BL body length; CBC, cannon bone circumference; CD, chest depth; CW, chest width; RC, rump circumference; RW, rump width; SW, scapula width; WW, waist width.

Above the diagonal are the phenotypic correlations and below the diagonal are the genetic correlations.

**Table S2. Distribution of SNPs and effective SNPs after quality control**

| **Chromosome** | **No. SNPs** | **Average distance(kb)^1^** | **No. effective SNPs** |
| --- | --- | --- | --- |
| 1 | 5155 | 61.17 | 1268 |
| 2 | 2122 | 76.61 | 436 |
| 3 | 1659 | 87.27 | 580 |
| 4 | 2903 | 49.42 | 823 |
| 5 | 1776 | 62.79 | 650 |
| 6 | 1505 | 104.83 | 563 |
| 7 | 2838 | 47.49 | 812 |
| 8 | 1770 | 83.89 | 585 |
| 9 | 2080 | 73.88 | 654 |
| 10 | 1094 | 72.31 | 481 |
| 11 | 1478 | 59.33 | 545 |
| 12 | 893 | 71.21 | 409 |
| 13 | 2860 | 76.45 | 812 |
| 14 | 3150 | 48.84 | 819 |
| 15 | 2025 | 77.87 | 694 |
| 16 | 1264 | 68.75 | 458 |
| 17 | 1314 | 53.05 | 517 |
| 18 | 901 | 67.95 | 383 |
| X | 668 | 216.00 | 550 |
| Y | 1 |  |  |
| 0^2^ | 10792 |  |  |
| Total | 48238 |  |  |

^1^ Derived from *Sus scrofa* Build 10.2.

^2^These SNPs are not assigned to any chromosomes.

**Table S3. Genome – wide significant SNPs of BH, BL, CBC, and RC**

| SNP | Chr | Pos1 | CBC | BL | BH | RC | CD |  |
| --- | --- | --- | --- | --- | --- | --- | --- | --- |
| M1GA0026359 | 0 | 0 | 1.45E-06 |  | 5.74E-07 |  |  |  |
| MARC0012864 | 0 | 10216180 | 1.94E-07 | 3.0542E-07 | 0.0000034 |  |  |  |
| ALGA0109063 | 0 | 10216393 | 1.94E-07 | 3.0542E-07 | 0.0000034 |  |  |  |
| ASGA0033028 | 0 | 126254981 | 1.07E-07 | 1.23205E-07 | 6.7E-07 |  |  |  |
| MARC0077640 | 0 | 133782819 | 1.99E-10 | 1.3463E-10 | 2.21E-08 | 9.92E-08 |  |  |
| H3GA0005226 | 1 | 304694455 |  |  |  |  | 2.70E-06 |  |
| INRA0024039 | 7 | 20825348 | 1.85E-06 |  |  |  |  |  |
| DRGA0007323 | 7 | 21629050 | 7.23E-07 | 1.46549E-06 | 3.68E-06 |  |  |  |
| ALGA0039319 | 7 | 21663626 | 3.03E-07 | 2.83602E-07 | 0.0000008 |  |  |  |
| ALGA0039341 | 7 | 21767963 | 6.48E-07 |  |  |  |  |  |
| ALGA0039400 | 7 | 22503194 | 1.77E-06 |  |  |  |  |  |
| ALGA0039474 | 7 | 24070676 | 1.94E-06 |  |  |  |  |  |
| ALGA0039477 | 7 | 24096043 | 2.53E-06 |  |  |  |  |  |
| ALGA0039611 | 7 | 26707358 | 3.69E-06 | 2.86157E-06 |  |  |  |  |
| ALGA0039628 | 7 | 27373527 |  | 4.01296E-06 |  |  |  |  |
| ISU10000867 | 7 | 27550218 |  | 3.15795E-06 |  |  |  |  |
| UMB10000108 | 7 | 27724510 | 1.70E-06 | 5.50363E-07 |  |  |  |  |
| H3GA0020450 | 7 | 27855827 |  | 3.69255E-06 |  |  |  |  |
| ASGA0032063 | 7 | 28068019 |  | 1.74711E-07 |  |  |  |  |
| ASGA0032174 | 7 | 31045816 |  | 2.09816E-06 |  |  |  |  |
| ALGA0039921 | 7 | 31237418 | 9.74E-09 | 2.45665E-08 | 2.16E-07 | 1.34E-06 |  |  |
| DRGA0007448 | 7 | 31628039 | 3.44E-09 | 2.69344E-08 | 1.08E-07 |  |  |  |
| ASGA0032302 | 7 | 32957768 | 8.02E-09 | 1.58645E-08 | 1.35E-07 | 2.4E-06 |  |  |
| ASGA0032313 | 7 | 33086096 | 7.78E-09 | 1.58525E-08 | 1.77E-07 | 2.5E-06 |  |  |
| ASGA0032322 | 7 | 33259683 | 5.79E-07 | 3.59687E-07 |  |  |  |  |
| ALGA0040120 | 7 | 33740960 | 1.22E-08 | 1.16762E-08 | 5.92E-07 |  |  |  |
| H3GA0020692 | 7 | 33790291 | 1.95E-09 | 1.03522E-08 | 1.95E-07 |  |  |  |
| MARC0079017 | 7 | 33876748 | 3.29E-07 | 6.25164E-08 | 1.18E-06 |  |  |  |
| ALGA0040148 | 7 | 33991469 | 6.76E-09 | 7.55042E-10 | 2.97E-07 |  |  |  |
| H3GA0020709 | 7 | 34064236 | 7.68E-07 | 1.24339E-07 | 2.05E-07 |  |  |  |
| H3GA0020739 | 7 | 34556148 | 2.84E-10 | 2.19969E-11 | 1.43E-08 | 2.67E-07 |  |  |
| H3GA0020752 | 7 | 34673190 |  | 1.22171E-06 |  |  |  |  |
| M1GA0009945 | 7 | 34691264 | 4.31E-07 | 4.63689E-08 | 1.63E-07 |  |  |  |
| M1GA0009960 | 7 | 34743041 | 3.89E-07 | 4.28624E-08 | 1.42E-07 |  |  |  |
| H3GA0020765 | 7 | 34755602 | 1.57E-10 | 1.12023E-11 | 1.3E-08 | 1.03E-07 |  |  |
| ALGA0040220 | 7 | 34776047 | 3.89E-07 | 4.28624E-08 | 1.42E-07 |  |  |  |
| MARC0058766 | 7 | 34803564 | 1.84E-10 | 1.29637E-11 | 1.74E-08 | 1.25E-07 |  |  |
| ALGA0040227 | 7 | 34835986 | 4.72E-07 | 4.81053E-08 | 1.77E-07 |  |  |  |
| ALGA0040243 | 7 | 34873838 | 3.89E-07 | 4.28624E-08 | 1.42E-07 |  |  |  |
| ASGA0032524 | 7 | 34925816 | 2.63E-06 |  |  |  |  |  |
| ALGA0040260 | 7 | 35002839 | 8.64E-09 | 1.44552E-09 | 8.25E-08 | 3.23E-06 |  |  |
| ALGA0040263 | 7 | 35017672 | 8.64E-09 | 1.44552E-09 | 8.25E-08 | 3.23E-06 |  |  |
| ASGA0032536 | 7 | 35150544 | 8.64E-09 | 1.44552E-09 | 8.25E-08 | 3.23E-06 |  |  |
| MARC0033464 | 7 | 35177641 | 1.31E-11 | 8.93866E-12 | 2.01E-09 | 7.97E-08 |  |  |
| ASGA0032526 | 7 | 35251345 | 8.64E-09 | 1.44552E-09 | 8.25E-08 | 3.23E-06 |  |  |
| H3GA0020824 | 7 | 35332373 | 5.69E-09 | 2.05943E-08 | 1.17E-07 | 1.74E-06 |  |  |
| ASGA0032549 | 7 | 35356274 | 5.69E-09 | 2.05943E-08 | 1.17E-07 | 1.74E-06 |  |  |
| ASGA0032562 | 7 | 35530333 | 1.28E-09 | 6.11576E-09 | 2.08E-08 | 3.77E-06 |  |  |
| INRA0024805 | 7 | 35579961 | 1.44E-09 | 5.59151E-09 | 2.2E-08 | 3.15E-06 |  |  |
| ASGA0032571 | 7 | 35709335 | 1.38E-09 | 6.11576E-09 | 2.14E-08 | 2.96E-06 |  |  |
| M1GA0010006 | 7 | 35880196 | 5.28E-09 | 6.24305E-09 | 6.71E-08 | 2.13E-06 |  |  |
| MARC0039836 | 7 | 35935629 | 1.90E-10 | 1.32695E-10 | 2E-08 | 9.84E-08 |  |  |
| H3GA0020842 | 7 | 35959385 | 1.07E-09 | 1.83118E-09 | 3.18E-08 | 1.25E-06 |  |  |
| ASGA0032583 | 7 | 35973567 | 1.06E-08 | 1.25367E-08 | 1.45E-07 |  |  |  |
| H3GA0020849 | 7 | 36004578 | 1.99E-10 | 1.3463E-10 | 2.21E-08 | 9.92E-08 |  |  |
| H3GA0020846 | 7 | 36202231 | 1.06E-08 | 1.25367E-08 | 1.45E-07 |  |  |  |
| INRA0024809 | 7 | 36329680 | 1.24E-08 | 1.576E-08 | 1.6E-07 |  |  |  |
| ASGA0032595 | 7 | 36497507 | 5.17E-11 | 2.57073E-10 | 8.93E-09 | 2.13E-06 |  |  |
| ALGA0040331 | 7 | 36684494 | 1.24E-08 | 1.576E-08 | 1.6E-07 |  |  |  |
| ALGA0040386 | 7 | 37342413 |  | 2.79831E-06 |  |  |  |  |
| MARC0039406 | 7 | 37467931 |  | 3.20594E-06 |  |  |  |  |
| MARC0096194 | 7 | 37507074 |  | 3.20594E-06 |  |  |  |  |
| DIAS0004695 | 7 | 37531069 | 3.86E-06 | 6.58022E-07 |  |  |  |  |
| ALGA0040423 | 7 | 37731099 | 6.70E-08 | 4.03573E-08 | 2.07E-07 |  |  |  |
| ASGA0032705 | 7 | 38099549 | 5.09E-07 | 3.35975E-08 |  |  |  |  |
| MARC0061348 | 7 | 38213072 | 3.93E-06 | 2.43375E-07 |  |  |  |  |
| ALGA0040467 | 7 | 38400663 |  | 2.91412E-06 |  |  |  |  |
| DRGA0007508 | 7 | 38819938 | 3.93E-06 | 2.43375E-07 |  |  |  |  |
| DIAS0000130 | 7 | 39089506 | 3.73E-08 | 2.13094E-08 | 1.86E-07 |  |  |  |
| ALGA0040529 | 7 | 39275257 | 1.80E-06 | 4.06197E-07 | 4.81E-07 |  |  |  |
| DIAS0000369 | 7 | 39330435 | 7.30E-07 | 3.41875E-08 | 4.79E-07 |  |  |  |
| MARC0001110 | 7 | 39380322 | 3.20E-06 | 4.06197E-07 | 8.37E-07 |  |  |  |
| H3GA0020988 | 7 | 39592027 | 1.12E-07 | 8.31961E-08 | 4.06E-07 |  |  |  |
| ALGA0040570 | 7 | 39627760 | 3.19E-08 | 2.13785E-08 | 1.86E-07 |  |  |  |
| MARC0075587 | 7 | 39665300 |  | 2.17792E-06 |  |  |  |  |
| ASGA0032847 | 7 | 39969405 | 1.06E-07 | 6.09491E-07 | 0.0000027 |  |  |  |
| ASGA0032851 | 7 | 39985401 | 1.67E-08 | 6.2458E-08 | 1.49E-07 |  |  |  |
| ALGA0040629 | 7 | 40215039 |  | 1.50123E-06 |  |  |  |  |
| MARC0051108 | 7 | 40265147 | 5.94E-08 | 3.23737E-08 | 3.82E-07 |  |  |  |
| ALGA0040640 | 7 | 40398610 |  | 2.81322E-06 |  |  |  |  |
| M1GA0010112 | 7 | 40441888 | 1.09E-07 | 5.64756E-08 | 7.39E-07 |  |  |  |
| INRA0025056 | 7 | 40524179 | 1.01E-07 | 3.24681E-08 | 1.33E-07 |  |  |  |
| ALGA0040677 | 7 | 40679693 |  | 2.97775E-06 |  |  |  |  |
| ALGA0040695 | 7 | 40821762 |  | 2.48925E-06 |  |  |  |  |
| ASGA0032963 | 7 | 40847468 | 5.85E-08 | 1.78544E-08 | 2.03E-07 |  |  |  |
| ALGA0040717 | 7 | 41004531 | 2.48E-08 | 1.85951E-08 | 9.66E-08 |  |  |  |
| ALGA0040736 | 7 | 41184328 | 2.05E-07 | 3.3098E-07 |  |  |  |  |
| ALGA0040739 | 7 | 41202819 | 1.23E-07 | 9.08308E-08 | 7.04E-07 |  |  |  |
| H3GA0021132 | 7 | 41265270 | 6.32E-07 | 5.34422E-07 |  | 3.5E-06 |  |  |
| ALGA0040759 | 7 | 41391206 | 2.16E-07 | 2.77309E-07 | 1.37E-06 |  |  |  |
| ALGA0040772 | 7 | 41433961 | 6.74E-07 | 8.94275E-07 | 3.38E-06 |  |  |  |
| H3GA0021153 | 7 | 41762040 | 1.99E-07 | 1.80269E-07 | 8.91E-07 |  |  |  |
| ALGA0040786 | 7 | 41832022 | 1.26E-07 | 7.65862E-08 | 2.45E-07 |  |  |  |
| MARC0042115 | 7 | 41854212 | 2.84E-08 | 2.61219E-08 | 1.33E-07 |  |  |  |
| ALGA0040805 | 7 | 42070492 | 1.28E-07 | 6.6414E-08 | 6.03E-07 |  |  |  |
| ALGA0040824 | 7 | 42275330 | 8.32E-07 |  | 8.7E-07 |  |  |  |
| ALGA0040834 | 7 | 42489659 |  |  | 1.35E-06 |  |  |  |
| ASGA0033093 | 7 | 42787330 | 1.44E-07 | 1.06748E-07 | 6.36E-07 |  |  |  |
| MARC0027900 | 7 | 43062804 | 2.66E-07 | 1.73603E-07 | 5.21E-07 |  |  |  |
| ASGA0033095 | 7 | 43103102 | 1.51E-07 | 1.01375E-07 | 7.11E-07 |  |  |  |
| ALGA0040854 | 7 | 43127838 | 1.51E-07 | 1.01375E-07 | 7.11E-07 |  |  |  |
| ALGA0040856 | 7 | 43166101 | 1.62E-08 | 4.74069E-08 | 3.05E-07 | 3.66E-06 |  |  |
| ALGA0040857 | 7 | 43190587 | 1.64E-07 | 1.06373E-07 | 6.88E-07 |  |  |  |
| ASGA0033096 | 7 | 43205140 | 1.63E-07 | 1.14347E-07 | 7.34E-07 |  |  |  |
| ASGA0033098 | 7 | 43400560 |  | 3.46881E-06 | 3.36E-06 |  |  |  |
| MARC0042983 | 7 | 43656771 | 1.30E-07 | 7.39781E-08 | 7.41E-07 |  |  |  |
| INRA0025193 | 7 | 43759525 | 3.83E-07 | 3.93976E-06 |  |  |  |  |
| INRA0025194 | 7 | 43810375 | 9.39E-07 | 3.01123E-06 |  |  |  |  |
| DBNP0001311 | 7 | 43829354 | 1.30E-07 | 7.39781E-08 | 7.41E-07 |  |  |  |
| DIAS0000010 | 7 | 44026616 | 3.54E-07 | 2.37044E-07 | 0.0000021 |  |  |  |
| ASGA0033158 | 7 | 44637934 | 1.51E-07 | 1.03284E-07 | 8.95E-07 |  |  |  |
| ALGA0040921 | 7 | 44849233 | 1.69E-07 | 1.22878E-07 | 0.0000011 |  |  |  |
| H3GA0021272 | 7 | 45140942 | 3.30E-06 |  |  |  |  |  |
| DBMA0000241 | 7 | 45173179 | 8.55E-08 | 2.72426E-06 | 3.15E-06 | 1.45E-06 |  |  |
| ALGA0040937 | 7 | 45185966 | 9.37E-08 | 2.84834E-06 | 0.0000039 | 1.68E-06 |  |  |
| H3GA0021282 | 7 | 45305380 | 5.23E-07 | 1.80965E-06 |  | 8.43E-07 |  |  |
| MARC0076000 | 7 | 45338373 | 1.54E-06 | 3.63899E-06 |  | 1.34E-06 |  |  |
| M1GA0010230 | 7 | 45415054 | 4.38E-07 | 1.43853E-06 |  | 7.1E-07 |  |  |
| ALGA0040948 | 7 | 45470325 | 1.96E-06 |  |  |  |  |  |
| MARC0020021 | 7 | 45583203 | 4.17E-07 |  |  | 3.68E-06 |  |  |
| MARC0077571 | 7 | 46332094 | 1.09E-07 | 1.04474E-06 | 4.9E-07 |  |  |  |
| ASGA0033213 | 7 | 46516600 | 3.18E-06 |  | 0.0000014 |  |  |  |
| MARC0015432 | 7 | 47106377 | 1.62E-07 | 2.05188E-06 |  | 1.99E-06 |  |  |
| ASGA0033277 | 7 | 47414032 | 1.11E-07 | 2.35057E-06 |  | 6E-07 |  |  |
| ALGA0041094 | 7 | 48241835 | 7.55E-07 |  |  | 2.19E-06 |  |  |
| M1GA0010262 | 7 | 48567905 | 1.12E-07 | 1.4462E-06 | 4.01E-07 |  |  |  |
| ALGA0041431 | 7 | 52079544 | 1.85E-06 | 1.76428E-06 |  |  |  |  |
| DIAS0001425 | 7 | 53156608 | 3.64E-06 |  |  |  |  |  |
| ASGA0033695 | 7 | 53478145 | 3.50E-06 |  |  |  |  |  |
| MARC0037657 | 7 | 53494821 | 3.50E-06 |  |  |  |  |  |
| ALGA0041729 | 7 | 54164066 | 5.66E-07 |  | 3.12E-06 |  |  |  |
| DIAS0004279 | 7 | 54529860 | 6.06E-07 |  | 3.82E-06 |  |  |  |
| MARC0028399 | 7 | 57663438 | 1.11E-06 |  | 2.81E-06 |  |  |  |
| SIRI0001183 | 7 | 68149980 |  |  | 1.88E-06 |  |  |  |
| MARC0039911 | 7 | 134146665 | 1.19E-06 | 1.78529E-06 | 3.54E-06 |  |  |  |
| M1GA0011538 | 7 | 134236731 | 4.46E-07 | 6.01322E-07 | 9.16E-07 |  |  |  |
| MARC0093860 | 7 | 134275238 | 1.06E-06 | 1.38095E-06 | 3.24E-06 |  |  |  |
| MARC0060950 | 7 | 134313767 | 7.53E-07 | 1.3124E-06 | 0.0000018 |  |  |  |
| ALGA0046005 | 7 | 134683639 | 6.17E-07 | 1.16687E-06 | 1.12E-06 |  |  |  |

^1^ Derived from *Sus scrofa* Build 10.2.

**Table S4. Percentage of phenotypic variance explanation of BH**

| Trait | Chr | Marker | marker_p | markerR^2^ |
| --- | --- | --- | --- | --- |
| BH | 7 | MARC0033464 | 4.93E-28 | 0.189366 |
| BH | 7 | H3GA0020765 | 5.91E-26 | 0.176309 |
| BH | 7 | MARC0058766 | 1.24E-25 | 0.174538 |
| BH | 7 | H3GA0020739 | 7.61E-25 | 0.169251 |
| BH | 7 | H3GA0020842 | 1.36E-24 | 0.175643 |
| BH | 7 | ALGA0040148 | 2.37E-24 | 0.166094 |
| BH | 7 | MARC0039836 | 5.83E-24 | 0.163834 |
| BH | 7 | H3GA0020849 | 7.00E-24 | 0.163073 |
| BH | 7 | MARC0077640 | 7.00E-24 | 0.163073 |
| BH | 7 | ASGA0032595 | 1.47E-23 | 0.161005 |
| BH | 7 | H3GA0020692 | 8.03E-23 | 0.156465 |
| BH | 7 | ALGA0040120 | 4.95E-22 | 0.151551 |
| BH | 7 | MARC0079017 | 6.32E-22 | 0.150396 |
| BH | 7 | ALGA0040717 | 1.24E-19 | 0.135277 |
| BH | 7 | DIAS0000369 | 4.48E-19 | 0.13177 |
| BH | 7 | ASGA0032562 | 1.59E-18 | 0.128078 |
| BH | 7 | ASGA0032571 | 2.02E-18 | 0.127394 |
| BH | 7 | INRA0025056 | 2.06E-18 | 0.127738 |
| BH | 7 | ALGA0040856 | 2.24E-18 | 0.128895 |
| BH | 7 | INRA0024805 | 2.36E-18 | 0.127336 |
| BH | 7 | ALGA0040243 | 5.21E-18 | 0.124818 |
| BH | 7 | M1GA0009960 | 5.37E-18 | 0.124335 |
| BH | 7 | ALGA0040220 | 5.37E-18 | 0.124335 |
| BH | 7 | ASGA0032963 | 8.27E-18 | 0.123073 |
| BH | 7 | ALGA0040227 | 8.88E-18 | 0.123249 |
| BH | 7 | ASGA0032526 | 9.21E-18 | 0.12295 |
| BH | 7 | ALGA0040260 | 9.21E-18 | 0.12295 |
| BH | 7 | ALGA0040263 | 9.21E-18 | 0.12295 |
| BH | 7 | ASGA0032536 | 9.66E-18 | 0.123004 |
| BH | 7 | ASGA0032302 | 1.12E-17 | 0.123526 |
| BH | 7 | ASGA0032313 | 1.51E-17 | 0.121507 |
| BH | 7 | M1GA0009945 | 1.99E-17 | 0.120872 |
| BH | 7 | H3GA0020709 | 2.45E-17 | 0.119892 |
| BH | 7 | M1GA0010006 | 3.25E-17 | 0.119242 |
| BH | 7 | MARC0042115 | 3.66E-17 | 0.118892 |
| BH | 7 | DRGA0007448 | 6.53E-17 | 0.117186 |
| BH | 7 | ALGA0040805 | 7.92E-17 | 0.116433 |
| BH | 7 | MARC0027900 | 9.07E-17 | 0.116033 |
| BH | 7 | ALGA0040834 | 9.39E-17 | 0.115932 |
| BH | 7 | ASGA0033093 | 1.03E-16 | 0.115848 |
| BH | 7 | ALGA0039319 | 1.65E-16 | 0.114632 |
| BH | 7 | ASGA0033096 | 2.57E-16 | 0.113133 |
| BH | 7 | ASGA0033095 | 2.69E-16 | 0.112815 |
| BH | 7 | ALGA0040854 | 2.69E-16 | 0.112815 |
| BH | 7 | ALGA0040857 | 2.77E-16 | 0.112912 |
| BH | 7 | MARC0042983 | 3.06E-16 | 0.112432 |
| BH | 7 | DBNP0001311 | 3.06E-16 | 0.112432 |
| BH | 7 | M1GA0010262 | 3.39E-16 | 0.112486 |
| BH | 7 | ALGA0040529 | 3.73E-16 | 0.112199 |
| BH | 7 | H3GA0020824 | 4.23E-16 | 0.111825 |
| BH | 7 | ASGA0032549 | 4.23E-16 | 0.111825 |
| BH | 7 | ASGA0033158 | 6.85E-16 | 0.110043 |
| BH | 7 | ASGA0032851 | 8.31E-16 | 0.109818 |
| BH | 7 | ALGA0040921 | 9.47E-16 | 0.109083 |
| BH | 7 | ALGA0039921 | 1.35E-15 | 0.108207 |
| BH | 7 | DIAS0000130 | 1.36E-15 | 0.108182 |
| BH | 7 | ALGA0040786 | 1.38E-15 | 0.108127 |
| BH | 7 | H3GA0020846 | 1.45E-15 | 0.10798 |
| BH | 7 | ASGA0032583 | 1.45E-15 | 0.10798 |
| BH | 7 | ALGA0040570 | 2.23E-15 | 0.106705 |
| BH | 7 | INRA0024809 | 2.55E-15 | 0.106468 |
| BH | 7 | ALGA0040331 | 2.55E-15 | 0.106468 |
| BH | 7 | DRGA0007323 | 4.08E-15 | 0.104892 |
| BH | 7 | SIRI0001183 | 5.78E-15 | 0.104179 |
| BH | 7 | MARC0001110 | 6.76E-15 | 0.103382 |
| BH | 7 | ALGA0040759 | 9.00E-15 | 0.102683 |
| BH | 7 | MARC0051108 | 1.56E-14 | 0.100878 |
| BH | 7 | ALGA0040824 | 1.77E-14 | 0.100653 |
| BH | 7 | DIAS0000010 | 2.42E-14 | 0.105396 |
| BH | 7 | MARC0077571 | 2.54E-14 | 0.099874 |
| BH | 7 | ALGA0040423 | 2.57E-14 | 0.099367 |
| BH | 7 | ASGA0033213 | 2.93E-14 | 0.098972 |
| BH | 7 | M1GA0011538 | 6.78E-14 | 0.096592 |
| BH | 7 | ALGA0040739 | 8.69E-14 | 0.095689 |
| BH | 7 | M1GA0010112 | 1.16E-13 | 0.095434 |
| BH | 7 | MARC0028399 | 1.41E-13 | 0.094376 |
| BH | 7 | ASGA0033028 | 1.78E-13 | 0.093667 |
| BH | 7 | H3GA0020988 | 3.44E-13 | 0.091957 |
| BH | 7 | ASGA0033098 | 4.31E-13 | 0.09098 |
| BH | 7 | H3GA0021153 | 5.33E-13 | 0.093002 |
| BH | 7 | DBMA0000241 | 7.20E-13 | 0.089701 |
| BH | 7 | ALGA0040937 | 7.79E-13 | 0.089601 |
| BH | 7 | ALGA0040772 | 9.65E-13 | 0.088661 |
| BH | 7 | MARC0060950 | 1.35E-12 | 0.088489 |
| BH | 7 | ALGA0046005 | 1.77E-12 | 0.088084 |
| BH | 7 | MARC0039911 | 3.69E-12 | 0.084686 |
| BH | 7 | ASGA0032847 | 4.25E-12 | 0.08398 |
| BH | 7 | MARC0093860 | 4.82E-12 | 0.083729 |
| BH | 7 | ALGA0041729 | 6.15E-12 | 0.083248 |
| BH | 7 | DIAS0004279 | 6.79E-12 | 0.082542 |

**Table S5. Percentage of phenotypic variance explanation of BL**

| Trait | Chr | Marker | marker_p | markerR2 |
| --- | --- | --- | --- | --- |
| BL | 7 | MARC0033464 | 5.50E-39 | 0.25482947 |
| BL | 7 | ASGA0032595 | 5.64E-39 | 0.25476842 |
| BL | 7 | H3GA0020849 | 3.41E-36 | 0.23865747 |
| BL | 7 | MARC0077640 | 3.41E-36 | 0.23865747 |
| BL | 7 | MARC0039836 | 3.84E-36 | 0.23870383 |
| BL | 7 | H3GA0020765 | 1.06E-35 | 0.23577803 |
| BL | 7 | MARC0058766 | 1.54E-35 | 0.23516684 |
| BL | 7 | H3GA0020739 | 5.15E-35 | 0.23173004 |
| BL | 7 | DRGA0007448 | 6.76E-33 | 0.21943734 |
| BL | 7 | H3GA0020842 | 2.28E-32 | 0.22592646 |
| BL | 7 | H3GA0020692 | 6.89E-32 | 0.21335309 |
| BL | 7 | ASGA0032313 | 3.52E-31 | 0.20905196 |
| BL | 7 | ASGA0032302 | 5.65E-31 | 0.2096669 |
| BL | 7 | ALGA0039921 | 7.96E-31 | 0.20688899 |
| BL | 7 | ALGA0040120 | 1.33E-28 | 0.19347473 |
| BL | 7 | ALGA0040148 | 1.34E-28 | 0.19289055 |
| BL | 7 | DIAS0004695 | 5.24E-25 | 0.1705458 |
| BL | 7 | MARC0061348 | 1.27E-24 | 0.16783019 |
| BL | 7 | DRGA0007508 | 1.27E-24 | 0.16783019 |
| BL | 7 | ASGA0032571 | 1.73E-24 | 0.16722648 |
| BL | 7 | ALGA0039611 | 2.02E-24 | 0.16654102 |
| BL | 7 | ASGA0032705 | 2.04E-24 | 0.16650565 |
| BL | 7 | INRA0024805 | 2.16E-24 | 0.16711095 |
| BL | 7 | ASGA0032562 | 2.22E-24 | 0.16652582 |
| BL | 7 | ALGA0040717 | 5.29E-24 | 0.163854 |
| BL | 7 | MARC0079017 | 5.95E-24 | 0.16352531 |
| BL | 7 | H3GA0020824 | 1.61E-23 | 0.16123116 |
| BL | 7 | ASGA0032549 | 1.61E-23 | 0.16123116 |
| BL | 7 | M1GA0010006 | 5.32E-23 | 0.15762483 |
| BL | 7 | ASGA0032322 | 6.63E-23 | 0.15724678 |
| BL | 7 | ALGA0039628 | 8.26E-23 | 0.15638742 |
| BL | 7 | H3GA0020846 | 1.57E-22 | 0.15456677 |
| BL | 7 | ASGA0032583 | 1.57E-22 | 0.15456677 |
| BL | 7 | ALGA0040856 | 1.79E-22 | 0.1563695 |
| BL | 7 | ASGA0032963 | 1.99E-22 | 0.15366284 |
| BL | 7 | INRA0024809 | 2.91E-22 | 0.15306085 |
| BL | 7 | ALGA0040331 | 2.91E-22 | 0.15306085 |
| BL | 7 | ASGA0032174 | 1.20E-21 | 0.14856788 |
| BL | 7 | INRA0025056 | 1.76E-21 | 0.14816155 |
| BL | 7 | ASGA0032526 | 4.35E-21 | 0.14512557 |
| BL | 7 | ALGA0040260 | 4.35E-21 | 0.14512557 |
| BL | 7 | ALGA0040263 | 4.35E-21 | 0.14512557 |
| BL | 7 | ALGA0040805 | 4.37E-21 | 0.14489111 |
| BL | 7 | ASGA0032536 | 4.76E-21 | 0.14509173 |
| BL | 7 | ASGA0032851 | 2.11E-20 | 0.14081531 |
| BL | 7 | ALGA0039319 | 3.40E-20 | 0.13944604 |
| BL | 7 | ALGA0040759 | 5.33E-20 | 0.13814896 |
| BL | 7 | MARC0042115 | 9.14E-20 | 0.13637701 |
| BL | 7 | ASGA0033093 | 1.47E-19 | 0.1349966 |
| BL | 7 | DIAS0000130 | 2.46E-19 | 0.13350668 |
| BL | 7 | ALGA0040772 | 2.70E-19 | 0.13366139 |
| BL | 7 | ALGA0040570 | 2.74E-19 | 0.13319787 |
| BL | 7 | ASGA0033095 | 4.01E-19 | 0.1318895 |
| BL | 7 | ALGA0040854 | 4.01E-19 | 0.1318895 |
| BL | 7 | MARC0042983 | 4.24E-19 | 0.1317288 |
| BL | 7 | DBNP0001311 | 4.24E-19 | 0.1317288 |
| BL | 7 | ALGA0040857 | 4.40E-19 | 0.13182638 |
| BL | 7 | ASGA0033096 | 5.08E-19 | 0.13140564 |
| BL | 7 | ASGA0033158 | 7.88E-19 | 0.12993152 |
| BL | 7 | ALGA0040423 | 9.01E-19 | 0.12974192 |
| BL | 7 | ALGA0040921 | 9.69E-19 | 0.12932806 |
| BL | 7 | MARC0051108 | 1.66E-18 | 0.12795345 |
| BL | 7 | ALGA0040739 | 2.73E-18 | 0.12651307 |
| BL | 7 | MARC0027900 | 2.84E-18 | 0.12619416 |
| BL | 7 | ALGA0040786 | 3.19E-18 | 0.12605463 |
| BL | 7 | M1GA0010112 | 4.21E-18 | 0.12603205 |
| BL | 7 | DIAS0000369 | 4.30E-18 | 0.12518175 |
| BL | 7 | H3GA0020988 | 5.56E-18 | 0.12501429 |
| BL | 7 | ASGA0033028 | 6.48E-18 | 0.12417528 |
| BL | 7 | DIAS0000010 | 4.56E-17 | 0.12511632 |
| BL | 7 | H3GA0021153 | 7.71E-17 | 0.12028596 |
| BL | 7 | MARC0077571 | 1.13E-16 | 0.11610882 |
| BL | 7 | ALGA0040386 | 1.20E-16 | 0.11538921 |
| BL | 7 | DRGA0007323 | 1.27E-16 | 0.11521895 |
| BL | 7 | MARC0096194 | 1.59E-16 | 0.11455362 |
| BL | 7 | MARC0039406 | 2.01E-16 | 0.1136864 |
| BL | 7 | ALGA0040629 | 5.45E-16 | 0.11072631 |
| BL | 7 | ASGA0032847 | 1.65E-15 | 0.10777485 |
| BL | 7 | INRA0025193 | 1.68E-15 | 0.10736778 |
| BL | 7 | M1GA0010262 | 3.16E-15 | 0.10582604 |
| BL | 7 | ALGA0040467 | 3.24E-15 | 0.10608829 |
| BL | 7 | ALGA0041431 | 3.41E-15 | 0.10526229 |
| BL | 7 | ALGA0040695 | 3.58E-15 | 0.10511912 |
| BL | 7 | ALGA0040640 | 3.84E-15 | 0.1049094 |
| BL | 7 | INRA0025194 | 7.16E-15 | 0.10336912 |
| BL | 7 | ALGA0040736 | 1.06E-14 | 0.10250795 |
| BL | 7 | DBMA0000241 | 2.19E-14 | 0.1003264 |
| BL | 7 | ALGA0040243 | 2.45E-14 | 0.09967001 |
| BL | 7 | M1GA0009945 | 3.20E-14 | 0.09886133 |
| BL | 7 | ALGA0040937 | 3.52E-14 | 0.09904714 |
| BL | 7 | UMB10000108 | 3.76E-14 | 0.09821712 |
| BL | 7 | M1GA0009960 | 4.82E-14 | 0.09731843 |
| BL | 7 | ALGA0040220 | 4.82E-14 | 0.09731843 |
| BL | 7 | ALGA0040227 | 8.61E-14 | 0.09587107 |
| BL | 7 | H3GA0020709 | 1.46E-13 | 0.09397128 |
| BL | 7 | M1GA0011538 | 2.24E-13 | 0.09296998 |
| BL | 7 | H3GA0021132 | 3.80E-13 | 0.09136348 |
| BL | 7 | MARC0060950 | 3.87E-13 | 0.09233631 |
| BL | 7 | ALGA0046005 | 8.25E-13 | 0.09044366 |
| BL | 7 | ASGA0033277 | 9.64E-13 | 0.08967104 |
| BL | 7 | ALGA0040529 | 2.27E-12 | 0.08590972 |
| BL | 7 | MARC0076000 | 3.95E-12 | 0.08420649 |
| BL | 7 | MARC0093860 | 1.11E-11 | 0.08116021 |
| BL | 7 | MARC0001110 | 1.22E-11 | 0.08061661 |
| BL | 7 | MARC0039911 | 1.35E-11 | 0.08069016 |
| BL | 7 | M1GA0010230 | 2.85E-11 | 0.07837286 |
| BL | 7 | ASGA0032063 | 3.71E-11 | 0.07718413 |
| BL | 7 | H3GA0021282 | 7.51E-11 | 0.07610811 |
| BL | 7 | ISU10000867 | 3.75E-10 | 0.07035979 |
| BL | 7 | ASGA0033098 | 4.28E-10 | 0.0697215 |
| BL | 7 | H3GA0020450 | 1.31E-09 | 0.06622444 |
| BL | 7 | ALGA0040677 | 1.43E-08 | 0.05880626 |
| BL | 7 | MARC0075587 | 3.79E-08 | 0.05546082 |
| BL | 7 | M1GA0009951 | 2.97E-06 | 0.04160404 |
| BL | 7 | H3GA0020752 | 3.06E-06 | 0.04157458 |

**Table S6. Percentage of phenotypic variance explanation of CBC**

| Trait | Chr | Marker | marker_p | markerR2 |
| --- | --- | --- | --- | --- |
| CBC | 7 | H3GA0020842 | 1.77E-05 | 0.037727653 |
| CBC | 7 | MARC0039836 | 3.02E-04 | 0.026739059 |
| CBC | 7 | H3GA0020849 | 3.11E-04 | 0.026601215 |
| CBC | 7 | MARC0077640 | 3.11E-04 | 0.026601215 |
| CBC | 7 | MARC0033464 | 3.47E-04 | 0.026251936 |
| CBC | 7 | H3GA0020765 | 3.62E-04 | 0.026111966 |
| CBC | 7 | MARC0058766 | 3.71E-04 | 0.026068865 |
| CBC | 7 | H3GA0020739 | 3.85E-04 | 0.025913644 |
| CBC | 7 | ASGA0032595 | 4.30E-04 | 0.025549283 |
| CBC | 7 | ALGA0040148 | 7.80E-04 | 0.023611623 |
| CBC | 7 | ALGA0040120 | 7.91E-04 | 0.023642128 |
| CBC | 7 | H3GA0020692 | 9.13E-04 | 0.023135608 |
| CBC | 7 | ALGA0040717 | 0.001164625 | 0.022302973 |
| CBC | 7 | ASGA0032963 | 0.001641776 | 0.021181393 |
| CBC | 7 | MARC0079017 | 0.001843332 | 0.020802877 |
| CBC | 7 | H3GA0020824 | 0.001845991 | 0.020867108 |
| CBC | 7 | ASGA0032549 | 0.001845991 | 0.020867108 |
| CBC | 7 | ALGA0040805 | 0.002031237 | 0.02048546 |
| CBC | 7 | MARC0061348 | 0.00237163 | 0.019978623 |
| CBC | 7 | DRGA0007508 | 0.00237163 | 0.019978623 |
| CBC | 7 | ALGA0040759 | 0.002379555 | 0.020033926 |
| CBC | 7 | INRA0025056 | 0.002394601 | 0.020046472 |
| CBC | 7 | M1GA0010006 | 0.002856659 | 0.019401634 |
| CBC | 7 | ASGA0032571 | 0.003528656 | 0.018708528 |
| CBC | 7 | INRA0024805 | 0.003601009 | 0.018703881 |
| CBC | 7 | ASGA0032562 | 0.003601764 | 0.018641225 |
| CBC | 7 | ALGA0040772 | 0.00402464 | 0.018337568 |
| CBC | 7 | H3GA0020846 | 0.004146005 | 0.018179249 |
| CBC | 7 | ASGA0032583 | 0.004146005 | 0.018179249 |
| CBC | 7 | DIAS0004695 | 0.004157698 | 0.018170001 |
| CBC | 7 | INRA0024809 | 0.004497507 | 0.017941726 |
| CBC | 7 | ALGA0040331 | 0.004497507 | 0.017941726 |
| CBC | 7 | ASGA0032526 | 0.004594142 | 0.017842164 |
| CBC | 7 | ALGA0040260 | 0.004594142 | 0.017842164 |
| CBC | 7 | ALGA0040263 | 0.004594142 | 0.017842164 |
| CBC | 7 | ASGA0032536 | 0.004617911 | 0.017854803 |
| CBC | 7 | ASGA0032313 | 0.0083283 | 0.015886161 |
| CBC | 7 | ASGA0032302 | 0.008543867 | 0.01596092 |
| CBC | 7 | DIAS0000010 | 0.009306684 | 0.016477383 |
| CBC | 7 | DRGA0007448 | 0.0117534 | 0.014751693 |
| CBC | 7 | ALGA0039921 | 0.017649278 | 0.013411133 |
| CBC | 7 | ALGA0040423 | 0.018150609 | 0.013318708 |
| CBC | 7 | DIAS0000369 | 0.021816922 | 0.012711393 |
| CBC | 7 | ALGA0039319 | 0.024712104 | 0.012320333 |
| CBC | 7 | ASGA0032705 | 0.02711999 | 0.011972771 |
| CBC | 7 | ASGA0032322 | 0.028703416 | 0.011824804 |
| CBC | 7 | ALGA0040856 | 0.033343444 | 0.011481605 |
| CBC | 7 | M1GA0009960 | 0.036748331 | 0.010969979 |
| CBC | 7 | ALGA0040220 | 0.036748331 | 0.010969979 |
| CBC | 7 | H3GA0020709 | 0.036835098 | 0.010962191 |
| CBC | 7 | ALGA0040243 | 0.037647218 | 0.010926455 |
| CBC | 7 | M1GA0009945 | 0.037954215 | 0.010899544 |
| CBC | 7 | ALGA0040227 | 0.038318927 | 0.010867855 |
| CBC | 7 | ASGA0033093 | 0.039412624 | 0.010756676 |
| CBC | 7 | MARC0042983 | 0.040864238 | 0.010619339 |
| CBC | 7 | DBNP0001311 | 0.040864238 | 0.010619339 |
| CBC | 7 | ASGA0033095 | 0.041115816 | 0.010599064 |
| CBC | 7 | ALGA0040854 | 0.041115816 | 0.010599064 |
| CBC | 7 | ALGA0040857 | 0.04151006 | 0.010585116 |
| CBC | 7 | ASGA0033096 | 0.04151006 | 0.010585116 |
| CBC | 7 | ASGA0033158 | 0.043690194 | 0.010398419 |
| CBC | 7 | ALGA0040921 | 0.044122097 | 0.010365915 |
| CBC | 7 | ALGA0039611 | 0.047415412 | 0.010128022 |
| CBC | 7 | MARC0027900 | 0.05094348 | 0.00989079 |
| CBC | 7 | ASGA0032851 | 0.065727981 | 0.009078242 |
| CBC | 7 | MARC0042115 | 0.074479603 | 0.00864877 |
| CBC | 7 | ASGA0033028 | 0.090650303 | 0.008010444 |
| CBC | 7 | H3GA0021153 | 0.090779395 | 0.008253567 |
| CBC | 7 | ALGA0041431 | 0.094059723 | 0.007861508 |
| CBC | 7 | ALGA0040570 | 0.097413301 | 0.007758351 |
| CBC | 7 | DIAS0000130 | 0.098193448 | 0.00773188 |
| CBC | 7 | H3GA0021282 | 0.09933247 | 0.007810704 |
| CBC | 7 | M1GA0010230 | 0.101271427 | 0.007667766 |
| CBC | 7 | MARC0051108 | 0.106071715 | 0.007475729 |
| CBC | 7 | ALGA0040739 | 0.10922178 | 0.007378579 |
| CBC | 7 | M1GA0010112 | 0.109577148 | 0.007417226 |
| CBC | 7 | ALGA0040937 | 0.114657758 | 0.007265744 |
| CBC | 7 | DBMA0000241 | 0.115235339 | 0.007236809 |
| CBC | 7 | ALGA0040786 | 0.117114163 | 0.007146934 |
| CBC | 7 | MARC0020021 | 0.119113398 | 0.00712635 |
| CBC | 7 | H3GA0020988 | 0.132611136 | 0.006768032 |
| CBC | 7 | UMB10000108 | 0.155586046 | 0.006203276 |
| CBC | 7 | ASGA0033277 | 0.173330583 | 0.005933278 |
| CBC | 7 | ALGA0041094 | 0.198536706 | 0.00576785 |
| CBC | 7 | ALGA0040529 | 0.223977383 | 0.004999888 |
| CBC | 7 | ASGA0033213 | 0.225704448 | 0.004965986 |
| CBC | 7 | MARC0001110 | 0.24597031 | 0.0046798 |
| CBC | 7 | ALGA0040736 | 0.249637073 | 0.004653833 |
| CBC | 7 | M1GA0011538 | 0.291380905 | 0.004122547 |
| CBC | 7 | M1GA0010262 | 0.302731953 | 0.003995039 |
| CBC | 7 | ASGA0032847 | 0.302772675 | 0.00399459 |
| CBC | 7 | MARC0060950 | 0.310846196 | 0.003953037 |
| CBC | 7 | ALGA0046005 | 0.318865242 | 0.00388676 |
| CBC | 7 | H3GA0021132 | 0.327064236 | 0.003737049 |
| CBC | 7 | MARC0077571 | 0.329183962 | 0.003727953 |
| CBC | 7 | MARC0093860 | 0.37893529 | 0.003251046 |
| CBC | 7 | MARC0039911 | 0.387989627 | 0.003177384 |
| CBC | 7 | MARC0028399 | 0.395953751 | 0.0030989 |
| CBC | 7 | ASGA0032524 | 0.424291805 | 0.00286802 |
| CBC | 7 | ALGA0041729 | 0.42634746 | 0.002866257 |
| CBC | 7 | DIAS0004279 | 0.428861813 | 0.002832231 |
| CBC | 7 | DRGA0007323 | 0.451279958 | 0.002657557 |
| CBC | 7 | INRA0025193 | 0.458697405 | 0.002598836 |
| CBC | 7 | ALGA0039341 | 0.46357479 | 0.002572189 |
| CBC | 7 | INRA0025194 | 0.472425967 | 0.002508989 |
| CBC | 7 | ALGA0039400 | 0.503235922 | 0.002301697 |
| CBC | 7 | ALGA0039477 | 0.536011848 | 0.002086928 |
| CBC | 7 | ALGA0039474 | 0.539167686 | 0.002067303 |
| CBC | 7 | INRA0024039 | 0.540181436 | 0.00205758 |
| CBC | 7 | MARC0076000 | 0.571204024 | 0.001874318 |
| CBC | 7 | ALGA0040948 | 0.757698082 | 9.26E-04 |
| CBC | 7 | H3GA0021272 | 0.768730368 | 8.79E-04 |
| CBC | 7 | ALGA0040824 | 0.889837288 | 3.91E-04 |
| CBC | 7 | ASGA0033695 | 0.972637868 | 9.28E-05 |
| CBC | 7 | MARC0037657 | 0.972637868 | 9.28E-05 |
| CBC | 7 | DIAS0001425 | 0.982229823 | 6.03E-05 |

**Table S7. Percentage of phenotypic variance explanation of RC**

| Trait | Chr | Marker | marker_p | markerR2 |
| --- | --- | --- | --- | --- |
| RC | 7 | MARC0039836 | 9.41E-08 | 0.052674 |
| RC | 7 | H3GA0020849 | 1.04E-07 | 0.052259 |
| RC | 7 | MARC0077640 | 1.04E-07 | 0.052259 |
| RC | 7 | ALGA0039921 | 1.26E-07 | 0.051757 |
| RC | 7 | MARC0033464 | 1.73E-07 | 0.050667 |
| RC | 7 | ASGA0032313 | 2.94E-07 | 0.049058 |
| RC | 7 | ASGA0032302 | 3.21E-07 | 0.049256 |
| RC | 7 | H3GA0020765 | 4.50E-07 | 0.047621 |
| RC | 7 | ASGA0032595 | 4.51E-07 | 0.047618 |
| RC | 7 | MARC0058766 | 5.04E-07 | 0.047342 |
| RC | 7 | H3GA0020739 | 5.70E-07 | 0.046872 |
| RC | 7 | H3GA0020842 | 6.74E-07 | 0.04872 |
| RC | 7 | ASGA0032571 | 2.25E-05 | 0.035154 |
| RC | 7 | INRA0024805 | 2.34E-05 | 0.035143 |
| RC | 7 | ASGA0032562 | 2.79E-05 | 0.03447 |
| RC | 7 | M1GA0010006 | 3.39E-05 | 0.033836 |
| RC | 7 | H3GA0020824 | 3.54E-05 | 0.033752 |
| RC | 7 | ASGA0032549 | 3.54E-05 | 0.033752 |
| RC | 7 | ALGA0041094 | 1.02E-04 | 0.032341 |
| RC | 7 | ASGA0032526 | 1.37E-04 | 0.029313 |
| RC | 7 | ALGA0040260 | 1.37E-04 | 0.029313 |
| RC | 7 | ALGA0040263 | 1.37E-04 | 0.029313 |
| RC | 7 | ASGA0032536 | 1.41E-04 | 0.029262 |
| RC | 7 | ALGA0040856 | 5.05E-04 | 0.025447 |
| RC | 7 | MARC0076000 | 8.66E-04 | 0.023348 |
| RC | 7 | H3GA0021132 | 0.001445 | 0.021669 |
| RC | 7 | H3GA0021282 | 0.00151 | 0.021814 |
| RC | 7 | M1GA0010230 | 0.001534 | 0.021546 |
| RC | 7 | DBMA0000241 | 0.002443 | 0.020014 |
| RC | 7 | ALGA0040937 | 0.002574 | 0.019874 |
| RC | 7 | MARC0020021 | 0.003447 | 0.018879 |
| RC | 7 | ASGA0033277 | 0.012051 | 0.014892 |

**Table S8. Gene Ontology of 24 genes**

| Category | Term | PValue | Genes |
| --- | --- | --- | --- |
| GOTERM_BP_FAT | GO:0015711~organic anion transport | 0.04460591 | SLC26A8, SLC22A7 |
| GOTERM_MF_FAT | GO:0051539~4 iron, 4 sulfur cluster binding | 0.026636667 | PRIM2, MOCS1 |
| GOTERM_MF_FAT | GO:0051536~iron-sulfur cluster binding | 0.052613242 | PRIM2, MOCS1 |
| GOTERM_MF_FAT | GO:0051540~metal cluster binding | 0.052613242 | PRIM2, MOCS1 |

**Table S9. Chromosome-wide significant SNPs of BH, BL, CBC, and RC after conditioned GWAS**

| Trait | SNP | Chromosome | Position | P-value |
| --- | --- | --- | --- | --- |
| RC | ALGA0045743 | 7 | 129610297 | 1.34E-05 |
|  | MARC0008869 | 7 | 128915717 | 1.38E-05 |
|  | H3GA0023727 | 7 | 129019539 | 1.52E-05 |
|  | MARC0103720 | 7 | 129760771 | 1.61E-05 |
|  | DIAS0004546 | 7 | 129292704 | 3.12E-05 |
|  | ALGA0045703 | 7 | 129233585 | 3.14E-05 |
|  | M1GA0011231 | 7 | 129041116 | 3.49E-05 |
|  | ASGA0037076 | 7 | 128999444 | 5.38E-05 |
|  | ASGA0037068 | 7 | 128822974 | 5.52E-05 |
| CBC | INRA0015303 | 4 | 92049839 | 1.03E-05 |
|  | ASGA0020433 | 4 | 85250559 | 2.28E-05 |
|  | MARC0054004 | 4 | 85033158 | 2.40E-05 |
|  | ALGA0026158 | 4 | 85294108 | 2.40E-05 |
|  | MARC0002648 | 4 | 76033076 | 2.52E-05 |
|  | CASI0006794 | 4 | 85087382 | 2.52E-05 |
|  | ALGA0124518 | 4 | 85608923 | 3.01E-05 |
|  | ALGA0026189 | 4 | 86326309 | 3.27E-05 |
|  | SIRI0000865 | 4 | 85105668 | 3.29E-05 |
|  | MARC0032957 | 4 | 85653759 | 3.67E-05 |
|  | ASGA0021413 | 4 | 110552282 | 3.75E-05 |
|  | ALGA0026139 | 4 | 85151430 | 4.36E-05 |
|  | INRA0015057 | 4 | 83571400 | 4.43E-05 |
|  | ASGA0020462 | 4 | 87870799 | 4.43E-05 |
|  | ALGA0027115 | 4 | 105998663 | 4.44E-05 |
|  | ASGA0021253 | 4 | 106018138 | 4.54E-05 |
|  | DIAS0000484 | 4 | 109093503 | 4.64E-05 |
|  | ASGA0020466 | 4 | 87889676 | 5.17E-05 |
|  | ALGA0026246 | 4 | 90213506 | 5.22E-05 |
|  | ASGA0021451 | 4 | 111835994 | 5.33E-05 |
|  | ALGA0027408 | 4 | 111526933 | 5.48E-05 |
|  | MARC0079561 | 4 | 109319576 | 5.56E-05 |
|  | MARC0011217 | 4 | 109928627 | 5.82E-05 |
|  | MARC0018255 | 4 | 84479048 | 5.99E-05 |
| BL | ALGA0118125 | 4 | 105327717 | 3.31E-05 |

^1^ Derived from *Sus scrofa* Build 10.2.
