# Supplementary material for: Genome-Wide Association Studies Identify the Loci for 5 Exterior Traits in a Large White × Minzhu Pig Population
Source: PLoS One. 2014 Aug 4;9(8):e103766. doi: 10.1371/journal.pone.0103766 (PMC4121205; doi:10.1371/journal.pone.0103766)

**Figure S1:** The Manhattan plots for AC, CW, RW, SW and WW.


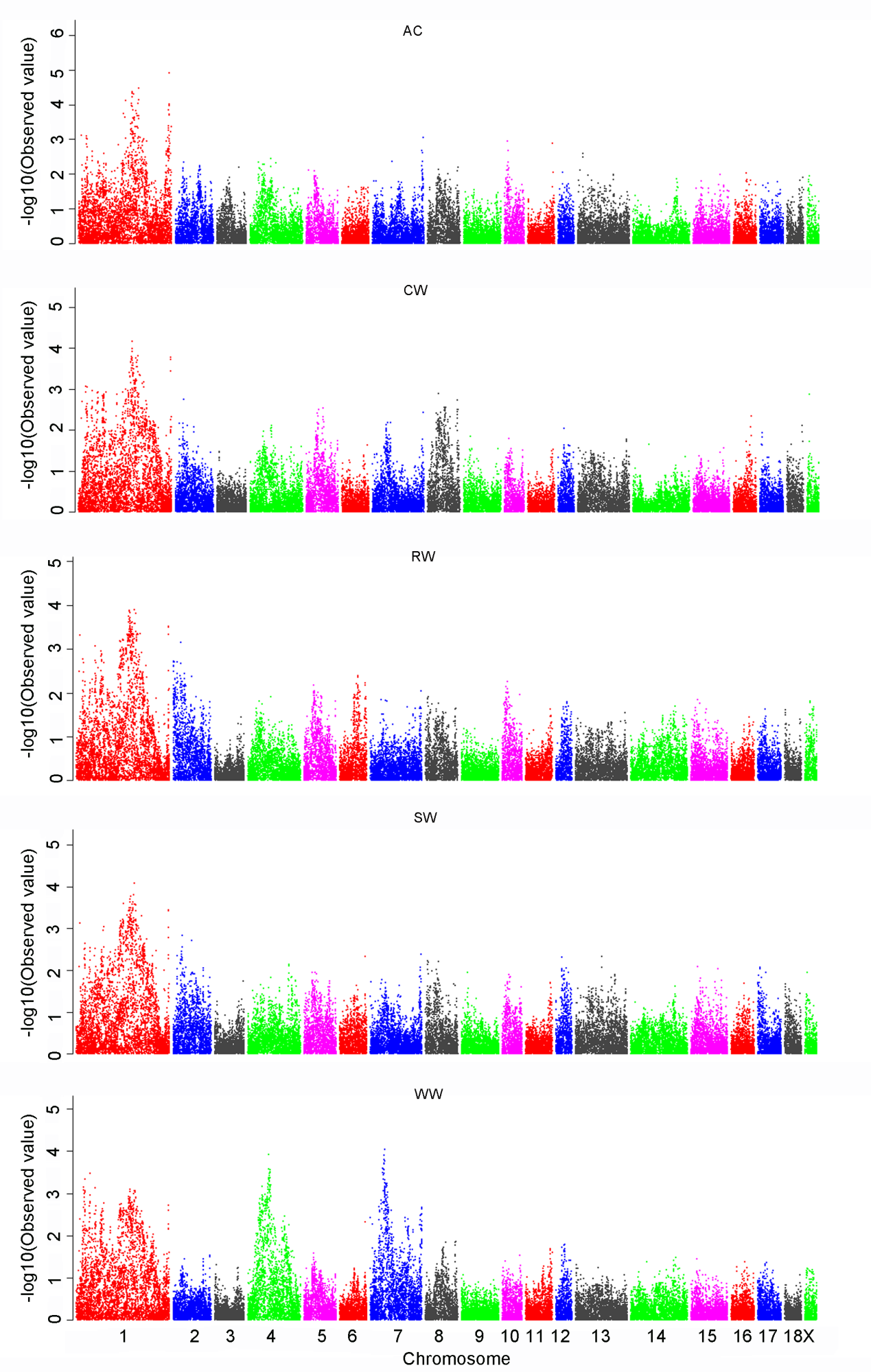


**Figure S2:** The Q-Q plots for AC, CW, RW, SW and WW.


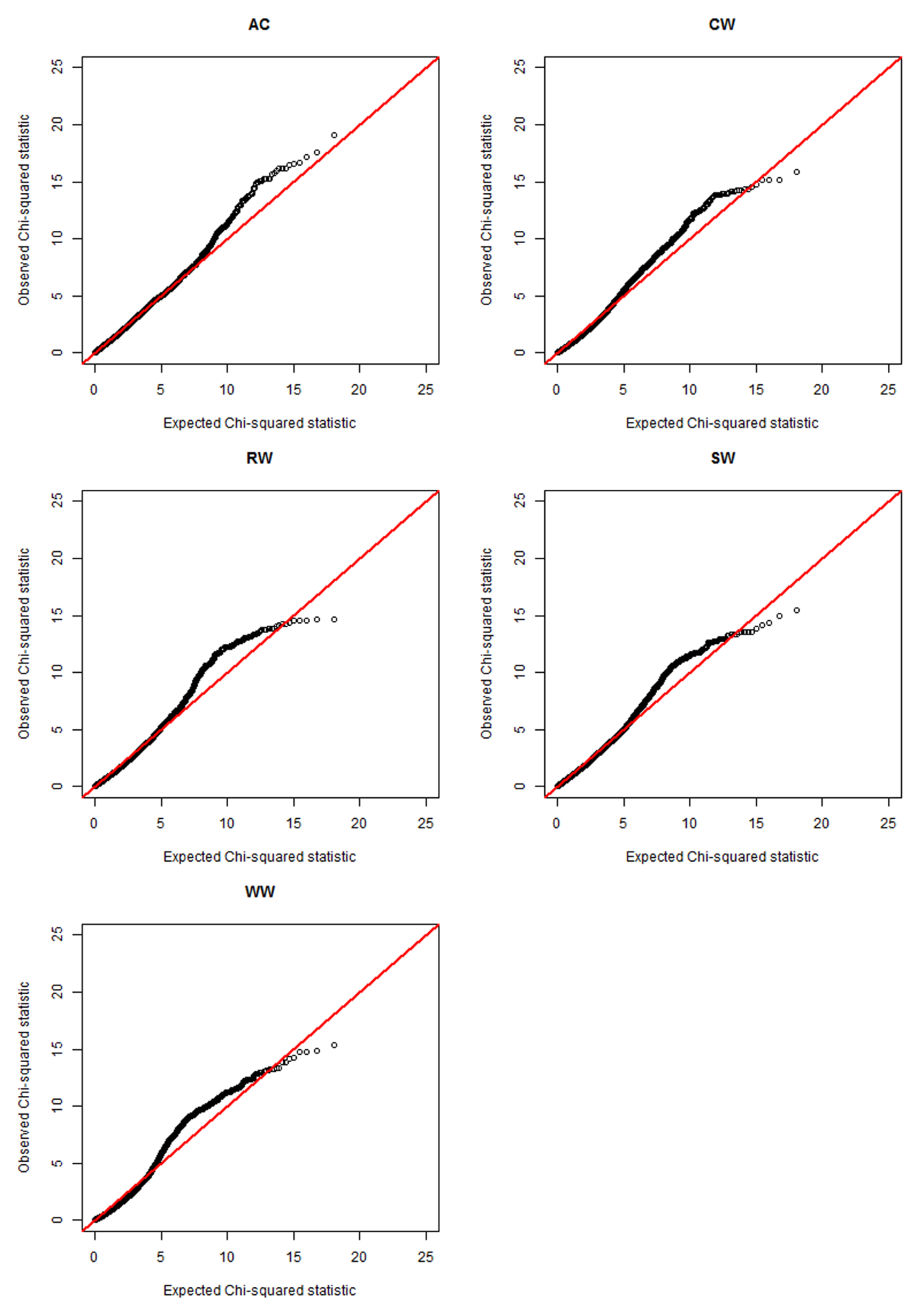


**Figure S3:** The Manhattan plots for conditioned GWAS for BH, BL, CBC, CD and RC.


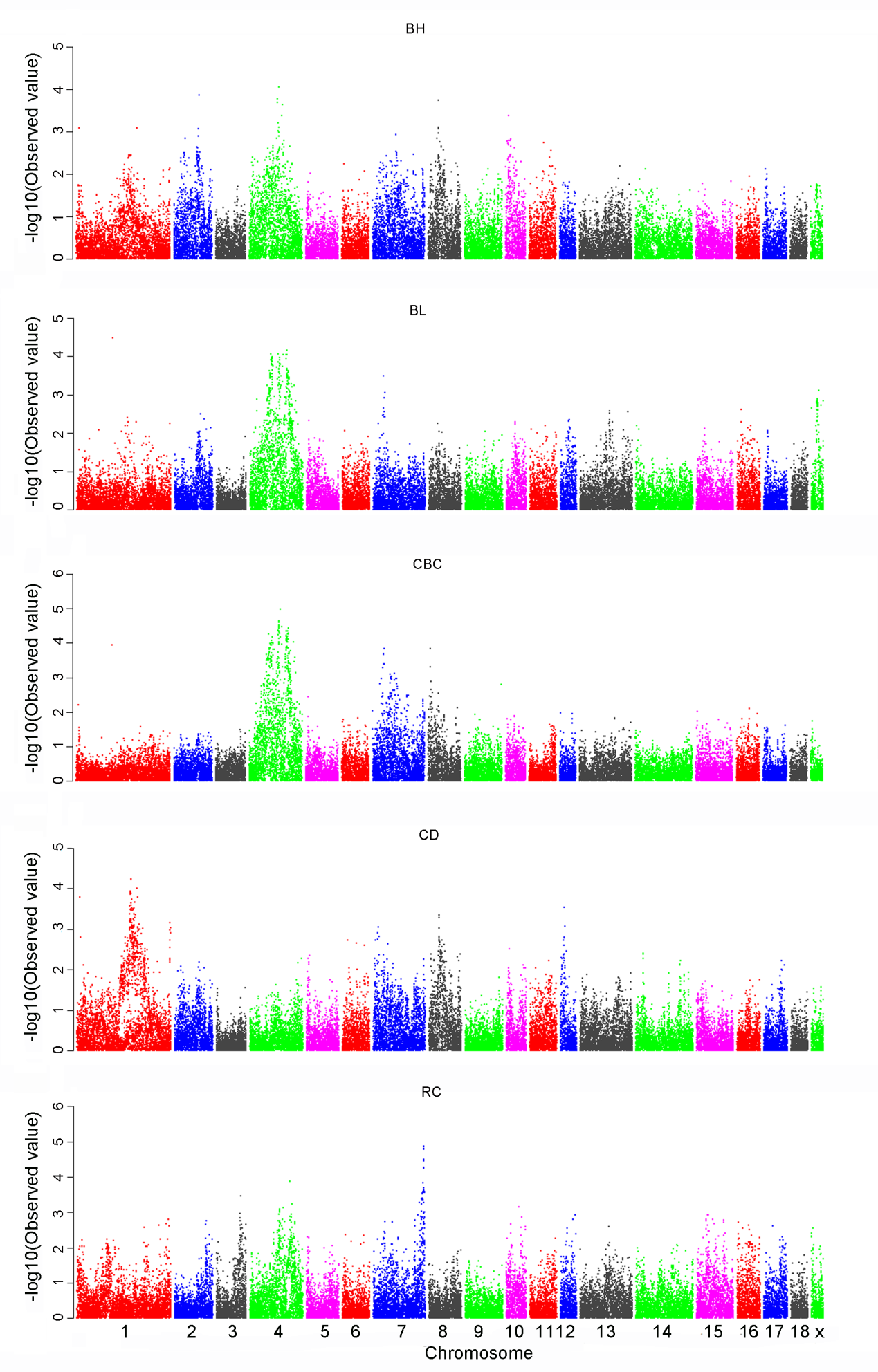

Supplement: File S2 — Figure S1. The Manhattan plots for AC, CW, RW, SW and WW. Figure S2. The Q-Q plots for AC, CW, RW, SW and WW. Figure S3. The Manhattan plots for conditional GWAS for BH, BL, CBC, CD and RC. (DOCX) [file pone.0103766.s002.docx]
